# D-dimer, disease severity, and deaths (3D-study) in patients with COVID-19: a systematic review and meta-analysis of 100 studies

**DOI:** 10.1038/s41598-021-01462-5

**Published:** 2021-11-08

**Authors:** Seshadri Reddy Varikasuvu, Saurabh Varshney, Naveen Dutt, Manne Munikumar, Shahir Asfahan, Paresh P. Kulkarni, Pratima Gupta

**Affiliations:** 1 Department of Biochemistry, All India Institute of Medical Sciences, Deoghar, 814142 India; 2 All India Institute of Medical Sciences, Deoghar, 814142 India; 3 Department of Respiratory Medicine, All India Institute of Medical Sciences, Jodhpur, 342005 India; 4 Department of Bioinformatics, ICMR-National Institute of Nutrition, Hyderabad, 500007 India; 5 Department of Biochemistry, Institute of Medical Sciences, Banaras Hindu University, Varanasi, 221005 India; 6 Department of Microbiology, All India Institute of Medical Sciences, Rishikesh, 249203 India

**Keywords:** Biochemistry, Biomarkers, Diseases, Risk factors

## Abstract

Hypercoagulability and the need for prioritizing coagulation markers for prognostic abilities have been highlighted in COVID-19. We aimed to quantify the associations of D-dimer with disease progression in patients with COVID-19. This systematic review and meta-analysis was registered with PROSPERO, CRD42020186661.We included 113 studies in our systematic review, of which 100 records (n = 38,310) with D-dimer data) were considered for meta-analysis. Across 68 unadjusted (n = 26,960) and 39 adjusted studies (n = 15,653) reporting initial D-dimer, a significant association was found in patients with higher D-dimer for the risk of overall disease progression (unadjusted odds ratio (uOR) 3.15; adjusted odds ratio (aOR) 1.64). The time-to-event outcomes were pooled across 19 unadjusted (n = 9743) and 21 adjusted studies (n = 13,287); a strong association was found in patients with higher D-dimers for the risk of overall disease progression (unadjusted hazard ratio (uHR) 1.41; adjusted hazard ratio (aHR) 1.10). The prognostic use of higher D-dimer was found to be promising for predicting overall disease progression (studies 68, area under curve 0.75) in COVID-19. Our study showed that higher D-dimer levels provide prognostic information useful for clinicians to early assess COVID-19 patients at risk for disease progression and mortality outcomes. This study, recommends rapid assessment of D-dimer for predicting adverse outcomes in COVID-19.

## Introduction

Ever since the emergence of COVID-19 in December, 2019, the severe acute respiratory syndrome coronavirus 2 (SARS-CoV-2) has spread rapidly across the globe^1^. With wide spectrum of symptoms, about 20–26% of patients with COVID-19 pneumonia become severe or critically ill, requiring hospitalization for respiratory support. With poor prognosis, the mortality rates vary from 26 to 61.5%^1–6^.

Early identification of patients at risk for disease progression is a major concern among clinicians, for developing management strategies in order to prevent mortality outcomes. Therefore, identification of better predictors of prognosis is of great clinical significance, and the need for prioritizing coagulation markers for prognostic abilities has been highlighted^5–7^. Several researchers have paid much attention to D-dimer, reporting its significant raise in severe cases and non-survivors, as compared to non-severe patients and survivors^5–27^. It has proposed that, as a marker of coagulation, increased D-dimer reflect hypercoagulability and thrombotic burden, guiding clinicians for using anticoagulation in COVID-19 patients^27–29^. Several studies have reported an increased D-dimer in positive relationship to disease severity, composite outcomes and high mortality events in COVID-19^30–55^. However, these individual studies are limited by sample size and reported different clinical outcomes based on unadjusted and/or adjusted models. Therefore, the available evidence on the prognostic information pertaining to D-dimer requires validation through meta-analysis. Further, the available reviews are of questionable quality (usually pooling of median and inter quartile ranges reported in included studies) or involve meta-analysis with less number of studies^56–60^.

In this study, we present a comprehensive meta-analysis to explore the prognostic use of D-dimer by the analysis of unadjusted and adjusted risk estimates (odds ratios) for disease severity, composite outcomes and mortality events. We also report the association of increased D-dimer with time-event-outcomes (unadjusted and adjusted hazards ratios) in COVID-19 patients. Further, the prognostic information of D-dimer was pooled for obtaining sensitivity, specificity, diagnostic odds ratio (DOR), and the area under curve (AUC) values for predicting COVID-19 disease progression.

## Methods

### Search strategy and selection criteria

The search for relevant literature was primarily conducted in PubMed, and then in the other databases such as Science Direct, Springer Author Mapper, Google Scholar, Scopus and Web of Science. The literature search was conducted using the keywords “COVID-19”, “nCoV-2019”, “nCoV”, “SARS-CoV-2”, “Novel Coronavirus”, “Severe Acute Respirator Syndrome Coronavirus-2” in combination with “Coagulation Dysfunction” and “D-dimer”. Considering the rapid growth in the COVID-19 research, the relevant databases (“LitCovid”, “CDC”, “WHO” and “NIH”), Coronavirus resource directories of major publishers (ELSEVIER, The Lancet, Springer and WILEY) and major journals (BMJ, NEJM, JAMA and the Major Respiratory Medicine journals) were searched. Additionally, the bibliographies of published articles were manually searched for potential literature. Two of the authors (SRV and PK) designed the search strategy for literature retrieval, and the other authors reviewed and verified the search strategy and retrieved literature. No filters/limits were applied during literature search. We followed PRISMA guidelines, and the protocol of this study was registered with PROSPERO, CRD42020186661.

Eligible studies had to report D-dimer results in COVID-19 patients. The eligible studies reported the direct effect sizes in the form of odds ratios of D-dimer or number of events in the form of 2 × 2 table (for predicting disease severity) and/or time-to-event outcomes in the form of hazards ratios of D-dimer for predicting deaths in COVID-19. Additionally, studies reporting direct ROC data or 2 × 2 tables were also eligible. The article types such as reviews, opinions, editorials, case-reports and studies not reporting D-dimer in association with COVID-19 severity and mortality were excluded. Three (SRV, PK, ND) independently screened (titles and abstracts) and reviewed the studies for their eligibility and any disagreements were resolved upon discussion with another author (SV) for consensus.

### Data extraction and definitions

The same authors (SRV, ND) involved in the literature search performed data extraction independently using the previously agreed-upon data extraction forms (MS word and colour coded-excel sheets). Any discrepancies were resolved via consensus with the other author (PK). The data extractions included: study author; country; study duration; age; male; female; outcomes (severity/mortality/CEP); number of higher D-dimer events (2 × 2 table data) in the severe versus non-severe and non-survival versus survival groups of COVID-19 patients; unadjusted and adjusted odds ratios and hazards ratios (with 95% CI levels) of D-dimer in relationship to COVID-19 disease severity and mortality; medication details; and percentages of comorbidities such as COPD, CVD, diabetes, hypertension.

COVID-19 diagnosis and severity definitions were according to the WHO interim guidance and/or the National health commission of China guidelines^61,62^. Severe COVID-19 was classified as having ARDS, oxygen saturation of ≤ 93%, need for ICU care or mechanical ventilation. The composite end point was defined as need for ICU care or mechanical ventilation or deaths. Mortality outcome was differentiated between non-survivals (deaths) versus survival (alive/discharged/recovered) COVID-19 patients. The time-to-event was defined as the time from hospitalization to ICU admission or death.

### Quality assessment and Statistical analysis

Two authors (SRV, ND) independently assessed the quality of eligible studies using the QUIPS tool^63^. Any disagreements were resolved by discussion with the Professor level third reviewer (SV). The quality assessment domains include: study participation; study attrition; prognostic factor measurement; outcome; confounding; statistical analysis and reporting. Based on these domains, the included studies were rated for risk of bias as ‘low’, ‘moderate’ or ‘high’.

The unadjusted and adjusted odds ratios and hazards ratios with 95% CI limits reported to describe the relationship of D-dimer with disease progression: severity; CEP; and mortality outcomes in COVID-19 patients were used. The data reported in 2 × 2 table format were used for obtaining unadjusted odds ratios. Considering the possible heterogeneity, random effects meta-analyses were conducted for conservative pooled-effect sizes for overall disease progression (severity + CEP + mortality) and sub-group analysis based on the individual ‘outcome type’ and ‘country’. Heterogeneity was assessed using the I-square statistic. The meta-regression and a one-study leave-out sensitivity analyses were performed to study the influence of certain variables and individual studies. Publication bias was tested using the funnel-plot asymmetry followed by Begg’s correlation and Egger’s regression tests. In case of a significant publication bias, Duval and Tweedie’s trim and fill method was used for obtaining adjusted values. We used Review Manager (Version 5.4) and Comprehensive Meta-analysis (Version 3) for the analysis of odds ratios and hazards ratios.

Further, meta-analysis of diagnostic test accuracy was conducted using relevant direct data and data from 2 × 2 tables to obtain pooled sensitivity, pooled specificity, positive- and negative-likelihood ratios, diagnostic odds ratios and AUC values using the random-effects DerSimonian-Laird method. The summary of receiver operating characteristic curves (SROC) was constructed with Moses linear model. Heterogeneity due to threshold and non-threshold effects was assessed by Spearman correlation analysis and the Cochran Q method with inconsistency (I^2^) test, respectively. These analyses were done using Meta-DiSc software (version 1.4).

## Results

**Figure 1:**
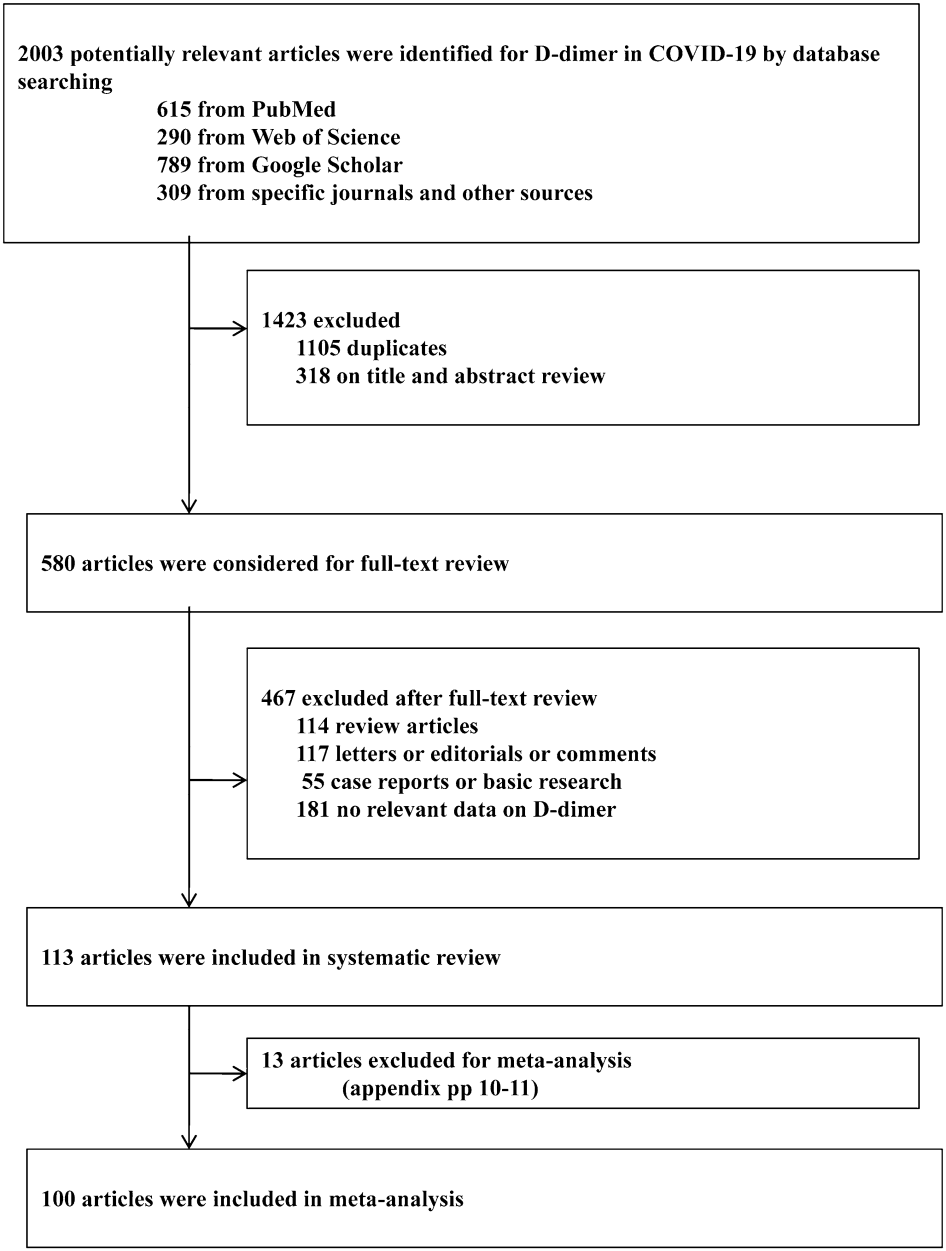
Study selection.

We identified a total of 2003 records by literature searching. Based on the initial screening of titles and abstracts, and after the removal of duplicates, 580 articles were considered further. Following the full-text review, 467 articles were removed, and the remaining 113 studies were included in systematic review (Fig. 1)^4–55,64–124^. Of these, 100 articles were finally included for meta-analysis (n = 38,310 with available D-dimer data)^4–55,64–111^. Across the included studies, the D-dimer levels were considered as ‘initial’ when measured within 24–48 h or the first measurement upon hospitalization. Whereas, the D-dimer levels were considered to be ‘dynamic’, for peak or longitudinal changes between different days, after initial assessment^7,37,40,43,45,55,64,86,88^. The adjusted factors vary across included studies (includes; age, sex, comorbidities, treatments and other lab variables) for obtaining independent effect sizes as adjusted odds and hazard ratios.

As we searched the literature for relevant studies of any design, majority of the included studies were retrospective cohorts, all included adult patients aged > 18 years (range 41–73 years), with the male and female % ranging from 36–91 to 8–64%, respectively. The proportions of any comorbidity across included studies range from 23.1 to 79.7%, with COPD (1–34%), diabetes (4.6–98.5%), and hypertension (14.2–79%). The proportions of deaths and recovery across reported studies were 1.4–74.07%, and 2.94–96.6%, respectively. Disease severity was compared between non-severe (mild-moderate) and severe to critical groups or requiring for ICU care or mechanical ventilation. Composite end point was defined as need for ICU care, mechanical ventilation and deaths. Time-to-event was defined as the time from hospital admission until the event or censoring. All studies were published in 2020: seventy-eight from China, 14 from USA, 10 from Italy, 4 from Spain, 3 from France, 2 from Turkey, one each from India, Iran and Europe. The criteria by WHO interim guidance or the national commission of China guidelines or laboratory confirmation by real-time polymerase chain reaction (RT-PCR) were used for COVID-19 across the studies. The main characteristics of the included (Appendix Table 1) and excluded studies (Appendix Table 2) and QUIPS assessments (Appendix Table 3) were presented in the Appendix (pp. 1–19).

**Figure 2:**
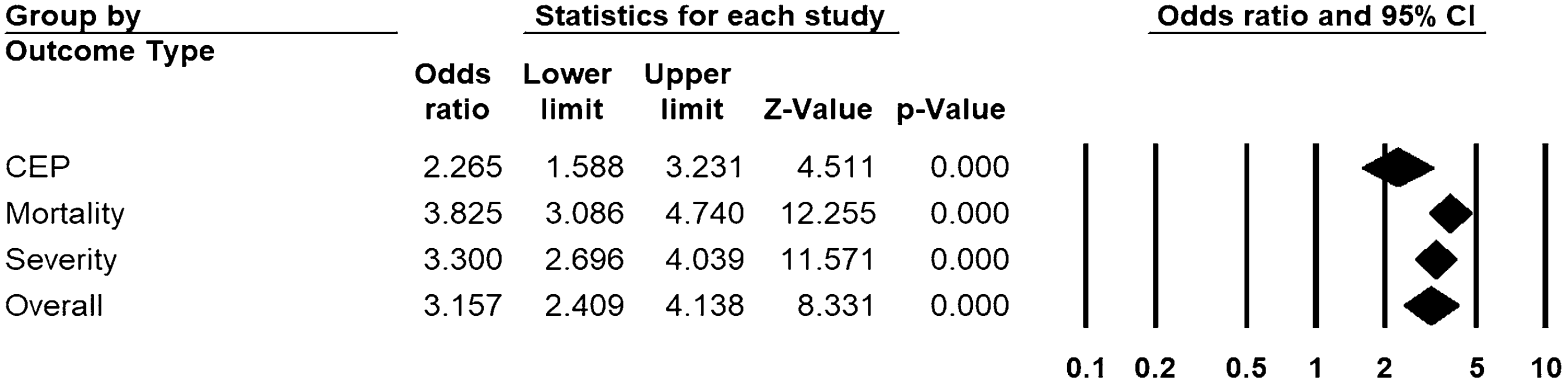
Pooled estimate of unadjusted odds ratios for the association of D-dimer with disease progression in patients with COVID-19.

**Figure 3:**
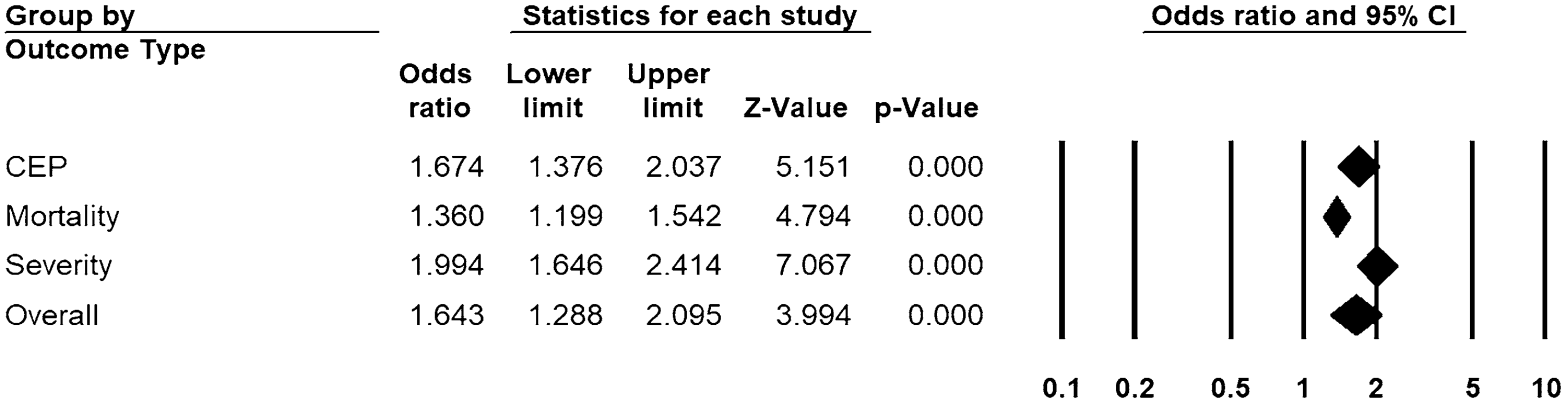
Pooled estimate of adjusted odds ratios for the association of D-dimer with disease progression in patients with COVID-19.

Across 68 unadjusted (n = 26,960) and 39 adjusted studies (n = 15,653) reporting initial D-dimer, a strong relationship was found in patients with higher D-dimers for the risk of overall disease progression (uOR 3.15, 95% CI 2.41 to 4.14, I^2^ = 92.3%, Fig. 2; aOR 1.64, 95% CI 1.29 to 2.10, I^2^ = 83.3%, Fig. 3). This pooled estimate corrected for publication bias, using trim and fill method, still showed a significant relationship (uOR 2.44, 95% CI 2.16 to 2.77; aOR 1.40, 95% CI 1.25 to 2.52). By sub-group analysis based on the outcome type, this association was found to be significant for disease severity (unadjusted studies 40, n = 15,358, uOR 3.30, 95% CI 2.67 to 4.04; adjusted studies 11, n = 4759, aOR 1.99, 95% CI 1.64 to 2.41), mortality (unadjusted studies 40, n = 15,613, uOR 3.82, 95% CI 3.08 to 4.74; adjusted studies 22, n = 9989, aOR 1.36, 95% CI 1.19 to 1.54), and CEP (unadjusted studies 11, n = 7004, uOR 2.26, 95% CI 1.58 to 3.23; adjusted studies 9, n = 4102, aOR 1.67, 95% CI 1.37 to 2.03) (Figs. 2 and 3, and Appendix pp. 20–23 for individual forest plots).

Across 11 unadjusted (n = 4702) and 7 adjusted observations on dynamic/peak D-dimer (n = 2063), a significant association was found in patients with higher D-dimers for the risk of overall disease progression (uOR 2.31, 95% CI 1.38 to 3.85, I^2^ = 93.9%; aOR 1.51, 95% CI 1.15 to 1.97, I^2^ = 87.8%). The strength of this association remained to be significant for mortality outcome (unadjusted studies 7, uOR 3.13, 95% CI 1.98 to 4.92; adjusted studies 3, aOR 1.51, 95% CI 1.01 to 2.27), (Appendix p. 24).

**Figure 4:**
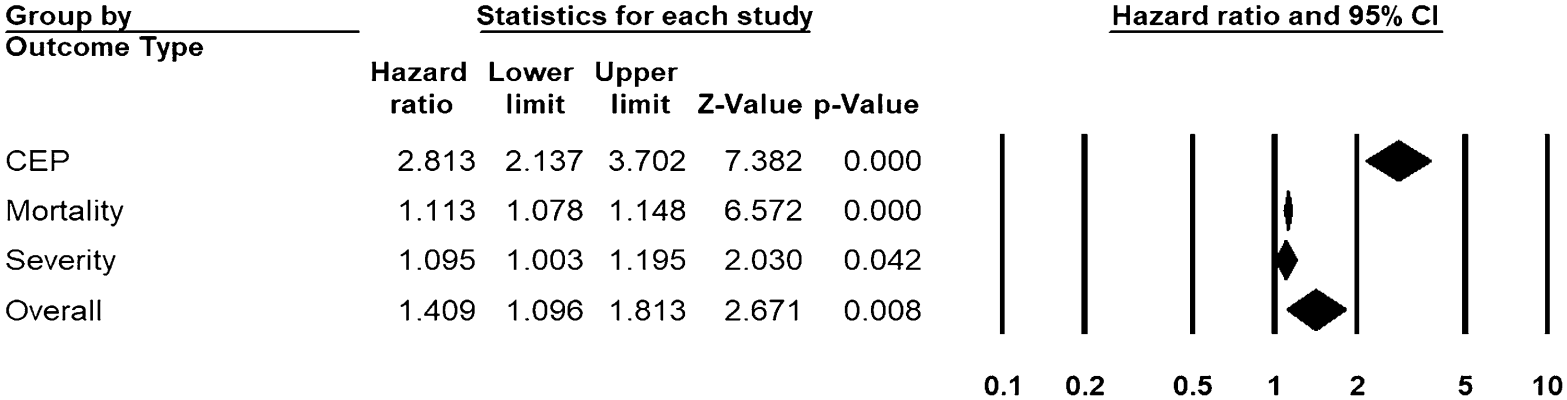
Pooled estimate of unadjusted hazard ratios for the association of D-dimer with disease progression in patients with COVID-19.

**Figure 5:**
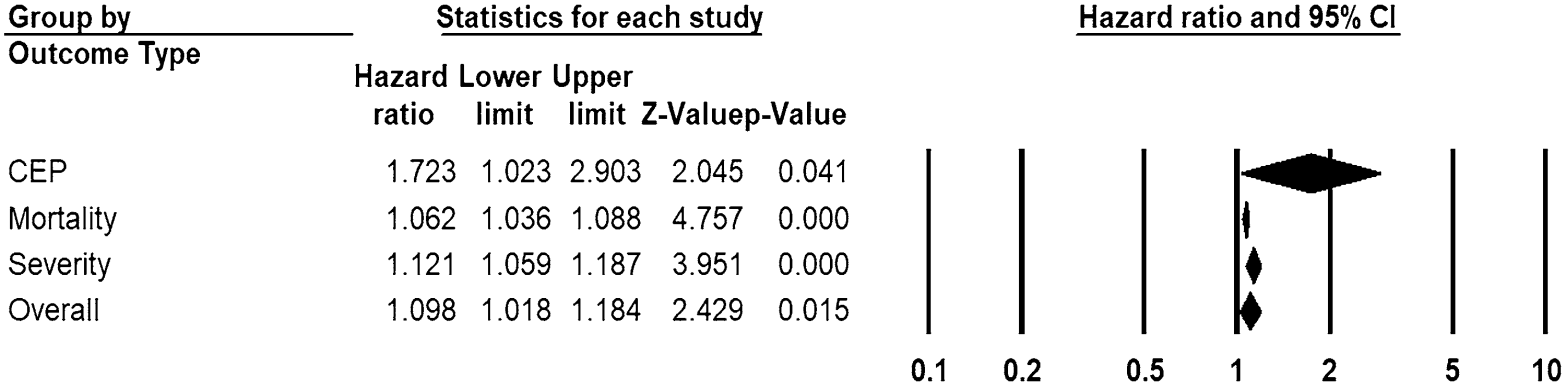
Pooled estimate of adjusted hazard ratios for the association of D-dimer with disease progression in patients with COVID-19.

Across 19 unadjusted (n = 9743) and 21 adjusted studies (n = 13,287) reporting time-to-event estimates, a strong association was found in patients with higher D-dimers for the risk of overall disease progression (uHR 1.41, 95% CI 1.10 to 1.81, I^2^ = 95.3%, Fig. 4; aHR 1.10, 95% CI 1.02 to 1.20, I^2^ = 91.4%, Fig. 5). This pooled estimate corrected for publication bias, using trim and fill method, still showed a significant relationship (uHR 1.08, 95% CI 1.04 to 1.12; aHR 1.07, 95% CI 1.04 to 1.09). By sub-group analysis based on the outcome type, this association was found to be significant for disease severity (unadjusted studies 3, n = 1621, uHR 3.30, 95% CI 2.67 to 4.04; adjusted studies 3, n = 1645, aHR 1.10, 95% CI 1.003 to 1.195), mortality (unadjusted studies 13, n = 8834, uHR 1.11, 95% CI 1.07 to 1.15; adjusted studies 15, n = 11,586, aHR 1.06, 95% CI 1.03 to 1.09), and CEP (unadjusted studies 3, n = 796, uHR 2.81, 95% CI 2.14 to 3.70; adjusted studies 2, n = 1121, aHR 1.72, 95% CI 1.02 to 2.90) (Figs. 4 and 5, and Appendix p. 25).

The higher D-dimers were found to be useful in predicting overall disease progression (studies 68, sensitivity 0.59, specificity 0.62, DOR 4.92, AUC 0.75), severity (studies 32, sensitivity 0.55, specificity 0.56, DOR 3.49, AUC 0.69), mortality (studies 32, sensitivity 0.64, specificity 0.66, DOR 7.20, AUC 0.79), and CEP outcomes (studies 4, sensitivity 0.75, specificity 0.55, DOR 4.70, AUC 0.93) (Table 1).

**Table 1:** Pooled estimates of sensitivity, specificity, diagnostic odds ratio, and AUC values of D-dimer for predicting disease progression.

	Severity	Mortality	CEP	Overall-disease progression
**Pooled sensitivity**				
n	32	32	4	68
I-Square	96.8	90.8	50.6	95.2
Pooled Sensitivity	0.55	0.64	0.75	0.59
CI	0.54–0.57	0.62–0.66	0.69–0.80	0.57–0.60
**Pooled specificity**				
n	32	32	4	68
I-Square	95.8	98.7	92.0	98.0
Pooled Specificity	0.56	0.66	0.55	0.62
CI	0.55–0.57	0.65–0.67	0.52–0.59	0.61–0.63
**Pooled PLR**				
n	32	32	4	68
I-Square	86.6	96.8	87.7	93.8
Pooled PLR	1.69	2.67	1.95	2.12
CI	1.51–1.89	2.19–3.26	1.36–2.80	1.91–2.35
**Pooled NLR**				
n	32	32	4	68
I-Square	89.2	67.3	58.2	85.1
Pooled NLR	0.54	0.48	0.47	0.50
CI	0.46–0.63	0.43–0.54	0.31–0.69	0.45–0.55
**Pooled DOR**				
n	32	32	4	68
I-Square	83.8	82.7	81	84.9
Pooled DOR	3.49	7.20	4.70	4.92
CI	2.66–4.58	5.23–9.90	2.01–10.97	4.00–6.06
Cochran-Q	190.92	179.32	15.75	444.52
p	0.00	0.00	0.001	0.00
**Threshold effect**				
Spearman’s correlation	0.55	0.61	− 0.20	0.49
p	0.001	0.00	0.80	0.00
**SROC**				
AUC (SE)	0.69 (0.02)	0.79 (0.02)	0.95 (0.12)	0.75 (0.01)
CI	0.67–0.72	0.77–0.81	0.80–1.05	0.73–0.77

AUC = area under curve. CEP = composite end points. CI = confidence interval. DOR = diagnostic odds ratio. NLR = negative likelihood ratio. PLR = positive likelihood ratio. SROC = summary receiver operating curves.

By meta-regression (data not shown), the models comprised of age and sex (model 1), comorbidities (model 2), deaths and recovery % (model 3) could be potential contributors of heterogeneity. By sub-group analysis based on the variable ‘country’; studies presenting wider CIs of ORs (one each from ‘India’ and ‘Iran’, and 7 from ‘Italy’). Similarly, one multi-country study from ‘Europe’ presenting wider CIs of HR could be a possible source of heterogeneity. However, we did a random-effects meta-analysis that assigns weight to each included study by incorporating between-studies variance. We further analysed the robustness of our findings by sensitivity analysis, revealing that no particular observation could significantly affect any of the pooled estimates. The country-wide subgroup analyses were shown in Appendix (pp. 26–27).

## Discussion

The findings of this meta-analysis provide the best comprehensive evidence that higher D-dimer was associated with disease severity and CEP in COVID-19. The mortality outcome was significantly associated with higher D-dimer levels. Importantly, our analysis suggests that D-dimer (initial and dynamic) is an independent prognostic marker as evidenced by the unadjusted and adjusted-OR estimates for disease progression. Our analysis also suggests that higher D-dimer (initial) exhibits strong and independent association with time-to-event outcome estimates (unadjusted and adjusted-HRs). Further, our findings indicate that higher D-dimer exhibit good predictive abilities as a prognostic marker of disease severity (AUC 0.69), CEP (0.93) and mortality outcomes (0.79).

In accordance with previous evidence, our results on the association of higher D-dimer with disease progression in COVID-19 support that severe patients are at higher risk of hypercoagulability^34,38,97,98^. Also, a large body of evidence shows that, the non-surviving COVID-19 exhibit significantly higher D-dimer levels, reflective of hypercoagulability status^4,5,13–15,64–66^. These results suggest that higher D-dimer levels in COVID-19 patients might indicate coagulopathy and thrombotic risk. Several mechanisms explain higher D-dimer and hypercoagulability in COVID-19^5,6,125–127^. Critically ill patients present with more severe hypoxia and lung injury. Severe and critical COVID-19 patients are presented with higher PAI-1 levels leading to impaired fibrinolytic and thrombus dissolution systems^5,125^. Hypoxaemia induced vasoconstriction leading to reduced blood flow and vascular occlusion, endothelial dysfunction, inflammation, major comorbidities such as hypertension and diabetes, old age, and prolonged bed rest are among the other factors^126^. Severe and critical COVID-19 patients are usually complicated by other comorbidities, organ dysfunctions and disseminated intravascular coagulation (DIC). Thrombotic and haemorrhagic events were common complications in non-survivors. As a marker of coagulation, and an important component of DIC, increased D-dimer is associated with the mortality outcome^5,6,127^.

A body of evidence suggests a correlation between markers of inflammation and coagulopathy^10,29^, cytokine storm lead to thrombus formation through platelet activation^37,128^. Higher D-dimer levels in COVID-19 patients suggestive of higher risk for disease progression may also indicate higher risk for thrombotic events. D-dimer has been reported to be an important prognostic factor for abnormal DLCO. For patients with raised D-dimer, pulmonary rehabilitation is recommended even in the absence of severe respiratory symptoms. The same study reported radiographic and physiological abnormalities in high proportion of patients 3 months after their discharge^129^. Hanif et al.^37^ found that none of the patients on anticoagulation showed thrombotic complications, highlighting the potential for early anticoagulation. Evidence shows that anticoagulation treatments were promising in reversing the procoagulant pattern^130^. Several studies have reported anticoagulation strategies for critical COVID-19 patients^131^. Recent studies show improved outcomes with anticoagulation in COVID-19 patients^37,54,79,93,132,133^. There is a need for further well controlled randomized trials to determine the clinical effectiveness.

Of note, hypercoagulability has been reported to occur at the early stages of COVID-19^5,37^, and procoagulant state is evidenced with micro- and macro-thrombi in autopsy studies^134^. Therefore, it is important to identify coagulation parameters for classifying high risk individuals for earliest possible intervention of coagulopathy. Varied D-dimer measurements with disease progression could provide predictive information to clinicians for early recognition of COVID-19 patients at risk for developing outcomes. This would also assist the concerned clinicians to develop anticoagulation therapeutic strategies, at the earliest to prevent disease progression to mortality. Our results suggest that D-dimer is a useful predictive marker for the severity of disease, CEP and mortality in COVID-19. This study can come handy for the clinicians to select high risk patients for their early management and save medical resources for the growing number of cases.

Our study has some strengths and limitations. Though we report the association of dynamic D-dimer levels with disease progression, we found only 11 and 7 observations for uOR and aOR, respectively. Therefore, further studies monitoring the dynamic profiles of D-dimer are still needed. The strong associations of initial D-dimer level with overall disease progression, severity, CEP, and mortality outcomes in COVID-19 patients (using large sample sizes), were similar between uOR and aOR estimates. Of note, we also report strong relationship of initial D-dimer with time-to-event outcomes, significant in both uHR and aHR estimates. The predictive abilities of initial D-dimer were promising with good DOR and AUC estimates for COVID-19 disease progression. The important limitation is the heterogeneity among the observational studies with retrospective design, which is inevitable in the meta-analysis of prognostic studies. Though we used random-effects model, assigning weight to each included study by incorporating between-studies variance, the clinical heterogeneity could not be completely ruled-out. The degree and diversity of severity, different comorbidities and treatment options, sample sizes and adjusted variables might have affected the clinical course and outcomes estimates. Owing to the retrospective nature of the studies, it is possible that D-dimer levels might have influenced anticoagulant treatment decisions, and vice versa. Even though participants with available D-dimer data were only included in the respective analysis of included studies, missing participants could potentially have introduced some bias. As most of the studies retrospectively extracted D-dimer data from the medical records of the patients during the hospital admissions, clear information on the measurement methods was not available in the included studies with varied D-dimer cut-offs across the studies. As there is no information as to why these cut-offs varied across studies, this study highlighting the use of D-dimer measurements for predicting disease severity, support the ISTH guidelines recommending the need for accurate D-dimer reporting in COVID-19^135^. Nevertheless, this study is the largest to comprehensively Meta-analyze D-dimer as a prognostic marker in association with disease progression in large sample size of COVID-19 patients. This study provides reliable evidence based on rigorous statistical analysis of unadjusted and adjusted estimates for outcomes risk along with the pooled diagnostic accuracy indices. Owing to this promising evidence on D-dimer, its further use in combination with other markers could be investigated further in well controlled studies.

## Implications for practice

Our study identified D-dimer as a promising prognostic factor for predicting disease severity, composite and mortality outcomes in COVID-19. The associations were significant in unadjusted and adjusted models. Further, higher D-dimer levels in patients with COVID-19 were associated with time-to-event hazard ratios. Our study supports dynamic monitoring of D-dimer levels for early risk assessment by the clinicians involved in the management of COVID-19 patients using anticoagulation strategies. This supports the use of D-dimer as a prognostic marker for early identification of covid-19 patients at high risk for adverse outcomes.

## Implications for future research

Owing to the evidence of inflammation and hyper coagulation in COVID-19^136,137^, the measurements of a routine coagulation marker, D-dimer provide reliable predictive information on disease progression. However, as different cut-offs were used across studies, there is a need to establish one universal cut-off point for predicting disease progression in COVID-19. Further, well controlled prospective studies investigating multi-marker strategies along with D-dimer could provide much useful information for predicting disease progression in COVID-19.

## Conclusion

In this meta-analysis, we identified all reported prognostic information on D-dimer for disease progression and mortality events in patients with COVID-19. Our results suggest that higher D-dimer levels in COVID-19 patients are significantly associated with disease progression. Higher D-dimer levels, indicative of hypercoagulability, are found to predict disease severity, composite outcomes and mortality events in both unadjusted and adjusted models of odds ratios and time-to-events hazards ratios. Further, the increased D-dimer could provide promising prognostic information for predicting disease progression in COVID-19. This study, recommends for rapid assessment of this coagulation marker, also support the ISTH guidelines for accurate D-dimer reporting in COVID-19. Owing to the growing number of coronavirus cases, our study provides valuable information for clinicians to early assess COVID-19 patients at risk for disease progression and mortality outcomes. Further prospective randomized studies are needed to confirm the results of this meta-analysis.

## Supplementary Information

Supplementary Information.
