# Supplementary material for: D-dimer, disease severity, and deaths (3D-study) in patients with COVID-19: a systematic review and meta-analysis of 100 studies
Source: Sci Rep. 2021 Nov 8;11:21888. doi: 10.1038/s41598-021-01462-5 (PMC8576016; doi:10.1038/s41598-021-01462-5)
Supplement: Supplementary file 1 — Supplementary Information. [file 41598_2021_1462_MOESM1_ESM.docx]

**Supplementary Appendix**

***Appendix Table 1:*** The Study Characteristics

| **Study/Country** | **Study type/**  **IEC** | **COVID-19 criteria/**  **RT-PCR** | **Duration/Follow-up** | **Total-(n) with D-dimer** | **Cut-off** | **Outcome** | **Statistics** | **Effect size** | **Reference** |
| --- | --- | --- | --- | --- | --- | --- | --- | --- | --- |
| Aloisio Elena et al., 2020/Italy | Retrospective observational/  Yes | Nasopharyngeal | Feb-Mar 2020 | 349 | >16,280 μg/L FEU (>4 X upper  reference limit) | Severity (ICU vs. Non-ICU) | ULR, MLR | OR | 8 |
| Aloisio Elena et al., 2020 (2)/Italy | Retrospective observational/  Yes | Nasopharyngeal | Feb-Mar 2020 | 131 | >16,280 μg/L FEU (>4 X upper  reference limit) | Mortality (Non-survivors vs. Survivors) | ROC | OR, DTA | 9 |
| Al-Samkari Hanny et al., 2020/USA | Multicenter-retrospective/  Yes | nasopharyngeal/  oropharyngeal  swab or sputum specimen | Mar-April 2020 | 400 | 1001-2500 ng/mL & > 2500 ng/mL | Critical vs. Non-critical | ULR, MLR | OR | 10 |
| Ayanian Shant et al., 2020/USA | Retrospective/Yes | US FDA approved testing criteria | Mar-May 2020/8 Days | 299 | ≥ 3  μg/ml | Severity (ICU admission, Intubation) & Mortality | ULR, | OR | 11 |
| Bao Changqian et al., 2020/China | Prospective follow-up/Yes | WHO interim guidance/Yes | Feb-Mar 2020/1-2 Weeks | 178 | > 1 μg/mL | Severe vs. Non-severe | Number of events | OR | 12 |
| Baycan OF et al., 2020/Turkey | Retrospective single-center /Yes | World Health Organization  interim guidance/Yes | April 15 and  April 30, 2020 | 143 | NA | In-hospital Mortality | MLR | MULTI-OR | 13 |
| Berger JS et al., 2020/USA | Retrospective/Yes | PCR-confirmed COVID-19/Yes | March 1, 2020 and April 08, 2020/through May 13, 2020. | 2377 | ≥230 ng/mL | CEP (critical illness: intensive care, mechanical ventilation,  discharge to hospice, or death)  & mortality | ULR & MLR | UNI and MULTI-OR for CEP and Mortality | 14 |
| Bhadade Rakesh et al., 2020/India | Observational cohort study/Yes | PCR-confirmed COVID-19/Yes | Study period 65 Days | 373 | 0.753 µg/mL (Mean) | Mortality | ULR & BLR | UNI & Independent-OR for Mortaity | 15 |
| Bi Xiaojie et al., 2020/China | Retrospective/Yes | Chinese management guideline for COVID-19 (version 6.0) | Jan-Feb 202023 Days | 113 | > 0.5 mg/L | Severity | Uni and Multivariate Cox & Kaplan–Meier analysis | UNI-HR | 16 |
| Bossini Nicola et al., 2020/Italy | Retrospective/  Yes | Lab-confirmed COVID-19/nasopharyngeal | Mar-April 2020/Min-7, Max-19, Median-11 Days | 53 | > 400 ng/mL | Severity (ARDS) and Mortality | ULR, MLR | UNI-OR | 17 |
| Cariou Bertrand et al., 2020/France | Multicentre observational/  Yes | Lab-confirmed COVID-19/Yes | Mar-April 2020/7 Days | 1317 | Increase of one SD | CEP (Intubation and/or death) and Mortality | ULR, MLR | UNI-OR | 18 |
| Cecconi Maurizio et al., 2020/Italy | Retrospective/  Yes | WHO/pharyngeal swab, bronchoalveolar lavage fluid or endotracheal aspirate | Feb-Mar 2020/20 Days | 239 | (per 1 μg/L increase), and ≥ 0.35 μg/L | CEP  (ICU transfer or death) | Uni and Multivariate Cox PH & Kaplan–Meier analysis | UNI-HR | 19 |
| Cen Yuan et al., 2020/China | Multicenter-consecutive cohort/Yes | WHO interim guidance and Interim Guidelines for COVID-19 of China (6th edition)/ nasal and pharyngeal swab | Feb 2020/28 Days | 443 | ≥0.5 mg/L | CEP  (Severe/critical or death) | Uni and Multivariate Cox PH | UNI and MULTI-HR | 20 |
| Chen Lei et al., 2020/China | Consecutive/Yes | Chinese management guideline for  COVID-19 (version 7)/ PCR testing in 1790 cases | Jan-April 2020/18 Days | 1859 | Log10 D-dimer per mg/l  increase | Mortality | Uni and Multivariate Cox | UNI and MULTI-HR | 21 |
| Chen Liu Ying et al., 2020/China | Retrospective/Yes | diagnosis and treatment plan for the new coronavirus in China/ nasal and pharyngeal  swab | Feb-Mar 2020/followed till discharged with recovery, or death. | 502 | 3.24 mg/L (Mean) | Mortality | Uni and Multivariate Cox PH & Kaplan–Meier analysis | UNI and MULTI-HR | 22 |
| Chen Ruchong et al., 2020/China | Retrospective/Yes | WHO interim guidance, national health commission of China | By Mar-2020 | 459 | > 0.5 μg/mL | Severity (M-S-C) and Mortality | Events on either side of the cut-off. Multivariate Cox. | CALC-OR. No-HR reported for D-D. | 23 |
| Chen Tao et al., 2020/China | Retrospective case series/Yes | national health commission of China (6^th^ Edition)/ Throat swab | Jan-Feb 2020/Until 28 Feb-2020 | 247 | > 21 μg/mL | Mortality | Events on either side of the cut-off. | CALC-OR. | 24 |
| Cheng Anying et al., 2020/China | Retrospective single-center/Yes | national health committee of China (Version 5)/Yes | Feb-Mar 2020 | 305 | ≥0.845μg/L | Mortality | Uni and Multivariate Cox & Kaplan–Meier analysis, ROC | UNI and MULTI-HR,  DTA | 25 |
| Chilimuri Sridhar et al., 2020/USA | Retrospective/Yes | guidelines committee in accordance with the Centers for Diseases Control and Prevention and the NY State DOH /nasopharyngeal swab | March 9–April 9, 2020 | 375 | > 1000 ng/mL | Mortality | Univariable and Multivariable regression | UNI and MULTI-OR | 26 |
| Coppelli Alberto et al., 2020/Italy | Retrospective/? | ? | March 20 to  April 30, 2020 | 271 | NA | In-hospital Mortality | unadjusted  and adjusted Cox proportional  hazards models & Kaplan-Meier analysis | UNI-HR | 27 |
| Cummings Matthew et al., 2020/USA | Prospective cohort-Two centers/Yes | WHO/ nasopharyngeal or oropharyngeal swab  samples. | March-April, 2020/Until April 28, 2020 so that each patient had at least 28 days of observation | 257 | per decile increase | Mortality | Cox proportional hazards & Kaplan–Meier analysis | UNI and MULTI-HR | 28 |
| Di Micco et al., 2020/Italy | Case-control/Yes | Confirmed cases of COVID-19 were divided in two groups according to the diagnosis of isolated  pneumonia or pneumonia with SARS. | Feb-Mar 2020 | 67 | > 500–700 μg/dL, | Severity (with/without SARS) | Events on either side of the cut-off. | CALC-OR | 30 |
| Dong Yalan et al., 2020/China | Retrospective/Yes | National Health  Commission of China (version 7)/Throat swab | February 10, 2020 to February 29, 2020 | 147 | 3 fold increase | Severity | ULR, MLR | UNI and MULTI-OR | 31 |
| Duan Jun et al., 2020/China | Retrospective-two centers/Yes | National Health Commission of China. / Nasophrygeal or anal swab, sputum | January 1st to February 29th, 2020/Patients were followed up to becoming non-severe to severe or discharge (Median: 4 Days) | 348 | > 0.14 mg/L | Severity | ROC | DTA | 32 |
| Feng Xiaobo et al., 2020/China | Prospective single-center/Yes | World Health Organization  interim guidance/ sputum and nasopharyngeal swabs s | Jan 23, 2020 to February 22, 2020 (28 days) | 114 | ≥0.5 vs. <0.5 mg/L | CEP-Poor outcomes (requiring mechanical  ventilation and deaths). | univariate and multivariate Cox  proportional hazard models | UNI & MULTI-HR for CEP | 33 |
| Gao Yong et al., 2020/China | Retrospective/Yes | WHO interim guidance/Yes | January 23, 2020 to February 2, 2020 | 43 | 0.28 μg/L | Severity | Logistic regression & ROC | Adj-OR, DTA | 34 |
| Ghaffari Rahbar M et al., 2020/Iran | Folow-up/Yes | Confirmed COVID-19/ Oro- or nasopharyngeal swabs | February 20, 2020 till April 15, 2020 | 19 | 0.5 μg/mL | Mortality | Events on either side of the cut-off. | CALC-OR | 35 |
| Gormez Selcuk et al., 2020/Turkey | Retrospective/Yes | PCR-confirmed COVID-19/Yes | March 15, to  April 15, 2020/The median follow-up  was 7 (range, 6–11) days. | 247 | from 0.3 to 0.9 µg/mL | CEP-Poor outcomes (need for intensive care  unit (ICU), mechanical ventilation, or occurrence of death) | penalized maximum likelihood  logistic regression analysis | MULTI-OR for CEP | 36 |
| Guan Wei-jie et al., 2020/China | Retrospective? Multi-center/Yes | WHO interim guidance/ nasal and pharyngeal swab | December 11, 2019, and January 29, 2020. | 560 | ≥ 0.5 mg/L | Severity and CEP (ICU/MV/Death) | Events on either side of the cut-off. | CALC-OR | 4 |
| Hanif Ahmad et al., 2020-OR/USA | Retrospective/Yes | PCR confirmed COVID-19/ nasopharyngeal swab | March 15 and April 14, 2020 | 921 | Initial mean 3190 ng/mL, Peak 6206 ng/mL | Severity (Mechanical ventilation) and Morality | UNI log regression | UNI-OR for severity and Mortality | 37 |
| Harmouch Farah et al., 2020/USA | Retrospective/Yes | NA | March 1-April 15, 2020 | 420 | ≥ 1 μg/mL | Severity (ICU care, MV), Mortality, and CEP | ULR, MLR | UNI-OR for ICU, MV, CEP and Mortality.  MULTI-OR for ICU, MV and CEP | 38 |
| Huang Hong et al., 2020/China | Retrospective single-center/Yes | Chinese Guidance for COVID-19 (Version 5)/ Throat swab | February 7 to March 27, 2020 | 158 | 0.47 μg/mL (Median) | Clinical outcomes (Severity; MV and ICU care). CEP?? | ULR | UNI-OR for poor clinical outcomes; severity, ICU care and MV (CEP??) | 39 |
| Li Chenghong et al., 2020/China | Observation cohort (consecutive)/Yes | PCR-confirmed cases | January 10 to  February 28, 2020/28 Days | 749 | > 1 mg/L | 28-day survival. | Univariate & Kaplan–Meier analysis | UNI-OR for mortality | 7 |
| Li Chenze et al., 2020/China | Retrospective/Yes | Guidelines on the Diagnosis and Treatment of COVID-19 used in  Wuhan/Yes | January 29  and April 1, 2020 | 1970 | > 0.5 μg/mL | Severity (Critical vs. Non-critical), Mortality | ULR & MLR | UNI-OR for Severity & Mortality.  MULTI-OR for mortality. | 40 |
| Li Guozhen et al., 2020/China | Retrospective/Yes | PCR-Confirmed COVID-19/Yes | January 24 to March 15, 2020 | 199 | Elevated D-D levels | Mortality | Multivariate analysis | MULTI-OR for Mortality | 41 |
| Li Kaiyan et al., 2020/China | Retrospective/Yes | WHO interim guideline/ throat-swab | January 31 to March 5, 2020/Until Mar 25. | 102 | > 0.5- ≤ 1 μg/Ml & > 1 μg/mL | Mortality | ULR, MLR | UNI-OR | 42 |
| Li Qiubai et al., 2020/China | Retrospective multi-center/Yes | WHO, National Health  Commission of China/ nasal and pharyngeal swabs | January 20, to April 4, 2020 | 1449 |  | Mortality | ULR, MLR | UNI and MULTI-OR | 43 |
| Li Tao et al., 2020/China | Retrospective/Yes | WHO interim guidance/nasal and pharyngeal swab | February 1 to March 31, 2020 | 80 | 0.7 mg/L (Mean) | Severity (Severe vs. Non-severe) | ULR, MLR | MULTI-OR | 44 |
| Li Yong et al., 2020/China | Multi-center/NA | National health commission of China (Version 7)/? | Day 1 through Day 10 | 279 | 1 μg/mL increment in D-dimer. | CEP (disease deterioration and deaths) | Multinomial logistic regression model. | MULTI-OR | 45 |
| Li Yumin et al., 2020/China | Retrospective two-center/Yes | WHO interim guidance/? | Dec 31, 2019, to Apr 5, 2020/Median (21 Days) | 132 | ≥ 0.5 μg/L,  ≥ 1.0 μg/L,  ≥ 1.5 μg/L | ICU entry and Mortality | ULR, MLR | UNI and MULTI-OR | 46 |
| Liao Danying et al., 2020/China | Retrospective multi-site/Yes | National Health Commission of China (version 7)/Throat swab | Jan 23 to Feb 23, 2020/Up to March 20, 2020 | 214 | > 2 mg/L | Mortality | Events on either side of the cut-off and MLR | UNI and MULTI-OR | 5 |
| Liu Fengjun et al., 2020/China | Retrospective/Yes | 6th version of national  diagnosis and treatment protocols of COVID-19 in  China/Yes | January 20, 2020 and February 3, 2020/28 Days | 134 | ROC, binarized by median. | Severe-event-free survival | logistic regression and Cox proportional hazard models, ROC | MULTI-HR and AUC | 47 |
| Liu Jiao et al., 2020/China | Retrospective single-center/Yes | WHO interim  Guidance, National Health Commission of China (Version 6)/ nasal and pharyngeal swab | December 29, 2019 to February 28, 2020/The final follow-up date was March 2, 2020 | 1102 | 0.5-1 μg/mL,  > 1 μg/mL | in-hospital deterioration (Non-severe to Severe) and death in severe cases. | ULR, MLR & Kalan-Meier analysis | UNI and MULTI-OR for severity and mortality, and MULTI-OR for mortality | 48 |
| Liu Jingmei et al., 2020/China | Retrospective/Yes | WHO interim guidance, Chinese National HealthCommittee (Version 3-5)/ nose swab and/or throat swab | February 9 to February 15, 2020/up to February 29, 2020 | 107 | ≥ 0.05 μg/mL | CEP (ICU admission, MV, Death) | ULR, MLR | UNI and MULTI OR for CEP | 49 |
| Liu Q et al., 2020/China | Retrospective single-center/Yes | PCR-Confirmed COVID-19/Yes | Between February 1, 2020 and March 13, 2020 | 336 | 1.56 μg/mL. | Mortality | ULR, MLR, ROC | UNI and MULTI-OR and DTA | 50 |
| Liu Qin et al., 2020/China | Retrospective/Yes | Confirmed COVID-19 cases/Yes | NA | 123 |  | CEP (ICU admission, MV, Death) | ULR, MLR | UNI and MULTI-OR for CEP | 51 |
| Liu Sheng-Ping et al., 2020/China | Retrospective/Yes | WHO interim guidance, Chinese management guideline (Version 7)/Yes | February 1, 2020 to  February 24, 2020/until  March 31, 2020. | 255 | > 1 mg/L | CEP (ICU admission or death) | MLR | MULTI-OR for CEP | 52 |
| Liu Tao et al., 2020/China | Retrospective/Yes | WHO interim guidance, National Health Commission of China (Version 5)/ nasal and  pharyngeal swab | January 21 to February 16, 2020 | 80 | ≥ 0.5 mg/L | Severity | Events on either side of the cut-off. | CALC-OR | 53 |
| Liu Xiaoyan et al., 2020/China | Retrospective analysis of a random cohort and Multi-center RCT/Yes | National Health Committee of China (Version 6)/ nasopharyngeal swabs | February 3 to March 8, 2020 | 124 | > 500 µg/L | Severity | Events on either side of the cut-off. | CALC-OR | 54 |
| Long Hui et al., 2020/China | /Yes | National Health Commission of China/Yes | January 18, 2020 to March 5, 2020 | 115 | ≥ 0.55 mg/L | Severity and Mortality | Events on either side of the cut-off.  ROC | CALC-OR and AUC | 55 |
| Maeda Tetsuro et al., 2020/USA | Retrospectivesingle-center cohort (consecutive)/Yes | PCR-confirmed COVID-19 cases/ nasopharyngeal swab | March 13 to March 31, 2020/through May 2, 2020 | 224 | NA | Severity (IMV) and Mortality | MLR, ROC | MULTI-OR and AUC | 64 |
| Mikami Takahisa et al., 2020/USA | Retrospective multi-center/ | PCR-confirmed COVID-19 cases / nasopharyngeal swab | March 13 to April 17, 2020 | 2984 | > 2 μg/mL | Severity (Ambulatory vs. hospitalized) and Mortality | Events on either side of the cut-off, Univariate and  Multivariate Cox models | UNI-OR for severity and mortality.UNI and MULTI-HR for Mortality. | 65 |
| Moreno-Pérez Oscar et al., 2020/Spain | Retrospective/Yes | COVID-19 cases on TCZ | March 12 to May 2, 2020/83 Days (The final date of follow up was June 9, 2020). | 77 | 0.8 mg/mL (Median) | Mortality, TCZ therapy | Logistic regression | UNI and MULTI-OR | 66 |
| Naymagon Leonard et al., 2020/USA | Retrospective/Yes | PCR-confirmed COVID-19/ nasopharyngeal swab | March 1, 2020 and April 1, 2020 (21 Days?) | 1065 | each 1 μg/ml increase in  admission D-dimer level.  (Median 1.39 μg/mL). | Severity (Invasive mechanical ventilation) & Mortality | univariable and multivariable Cox-proportional  hazards models | UNI & MULTI-HR for Intubation and Mortality | 29 |
| Pan Feng et al., 2020/China | Case-Control study/Yes | WHO, Chinese National  Diagnosis and Treatment Protocols/ | January 27, 2020 to March 19, 2020 | 124 | > 3.06 mg/L | Mortality | Bivariate and MLR | UNI-OR | 67 |
| Paranjpe Ishan et al., 2020/USA | Retrospective multi-center/ | PCR-confirmed COVID-19/ nasopharyngeal swab. | February 27 to April 2,  2020 | 282 | > 2 µg/mL | Severity (ICU vs No-ICU), Mortality | Events on either side of the cut-off | CALC-OR | 68 |
| Petrilli Christopher et al., 2020/USA | Prospective cohort single-center/ | / nasopharyngeal or oropharyngeal swab  specimens | March 1, 2020 to April 8, 2020/May 5, 2020 | 2356 | > 2500 µg/L | CEP (ICU, MV, Discharge to hospice or death), Mortality | Logistic regression | UNI and MULTI OR for CEP, MULTI-HR for mortality. | 69 |
| Qin Wei et al., 2020/China | Retrospective/ | National health commission of china (Version 7)/ | Dec 2019 to Feb 2020 | 582 | 0.5 mg/L (Median) | Mortality | ULR, MLR | MULTI-OR for Mortality | 70 |
| Rocio Laguna-Goya et al., 2020/Spain | Prospective cohort single-center/Yes | / nasopharyngeal or oropharyngeal swabs or sputum | March 10 and April 12, 2020/By April 20, 2020. | 501 | Per Unit Increase, and ROC cut-off (>1386 ng/mL) | Mortality | ULR, ROC | UNI-OR for Mortality, DTA | 71 |
| Sardu Celestino et al., 2020/Italy | Prospective/Yes | PCR-confirmed COVID-19 | February 10 and April 20, 2020 | 164 | 2.68 mg/mL (Median) | Mortality | Cox-regression analysis & Kaplan-Meier survival analysis | UNI and MULTI-HR for Mortality | 72 |
| Sciascia Savino et al., 2020/Italy | *pilot pro­spective open, single-arm multicentre/?* | PCR-Confirmed COVID-19 | At least for 14 Days | 63 | 3500 ng/mL | Mortality | MLR, & Kalan-Meier analysis | MULTI-HR | 73 |
| Shang Yufeng et al., 2020/China | Retrospective/Yes  (Training & Validation Cohorts) | WHO interim guidance, Chinese management guideline for COVID-19 (version7)/ nasal and pharyngeal swab specimens | January 1 to March27, 2020/up to March 31, 2020 | 452 | > 0.5 μg/mL | Mortality | Events on either side of the cut-off and Multivariate analysis | UNI and MULTI-OR for Mortality | 74 |
| Sisó-Almirall Antoni et al., 2020/Spain | Retrospective multi-centre/Yes | PCR-confirmed COVID-19/nasal and pharyngeal samples | February 29 to  April 4, 2020. | 165 | > 500 mg/L | CEP (ICU admission and death) | Events on either side of the cut-off and MLR | UNI and MULTI-OR for CEP | 75 |
| Smadja David et al., 2020/France | Prospective observational cohort study (Consecutive)/Yes | / nasopharyngeal  swabs | NA | 40 | ≥ 1000 ng/mL | Severity (ICU vs. Non-ICU) | Events on either side of the cut-off | UNI-OR for Severity | 76 |
| Somers EC et al., 2020/USA | Single-center cohort/? | /Yes | March 9 to April 20, 2020/through May 19, 2020 (Median 47 Days). | 154 | > 1.2 mg/dL | survival probability post-intubation (Time from intubation to death or censoring). &  CEP at Day 28 (Hospitialized, MV, Superinfection, Discharge, Death) | Cox proportional hazards models and proportional odds models-Univariate and Bivariate models | OR for CEP and HR for Mortality | 77 |
| Sun Ying et al., 2020/China | ? | WHO, National Health Commission of China (Version 7)/ throat swab or  sputum, urine, blood or stool | ? | 63 | 1.97 mg/L (Mean) | Severity (Moderate-severe-critical) | multivariate Cox regression, binary logistic regression | MULTI-OR for severity | 78 |
| Tang Ning et al., 2020/China | Retrospective (consecutive patients)/Yes | WHO, National Health Commission of China/Yes | January 1 to February 13, 2020/28 Days (up to  March 13, 2020). | 449 | > 0.5 µg/mL | Mortality | MLR | MULTI-OR for mortality | 79 |
| Tao Zheying et al., 2020/China | Retrospective single-centre/Yes | WHO interim guidance, Chinese National Health Committee (Version 7)/Yes | December 1, 2019 to March 20, 2020 | 222 | ≥ 0.5 mg/L | Severity (Non-severe vs. Severe) | ULR and MLR | UNI and MULTI-OR for Severity | 80 |
| Tian Jianbo et al., 2020/China | Retrospective multi-center/Yes | WHO, National Health Commission of China (Version 7)/Yes | Jan 13 and March 18, 2020/29 Days (until March 26, 2020) | 232 | 1.2 µg/mL (Median) | Severity (Non-severe vs. severe) | ULR and MLR | UNI and MULTI-OR | 81 |
| Volo T et al., 2020/Italy | Retrospective/Yes? | Confirmed-COVID-19/ nasal pharyngeal swab | 22nd February 2020 to 26th April 2020/50 Days | 23 | > 4 | Mortality | ULR and MLR.  Events on either side of the cut-off | UNI-OR for mortality.  Only a p-value (0.028) was reported for MULTI-OR | 82 |
| Wang Feng et al., 2020/China | Prospective?/Yes | Chinese National Health Commission / Throat-swab | January 2020 | 65 | > 0.5 μg/mL | Severity (Mild-severe-Extremely severe/Critical) | Events on either side of the cut-off | CALC-OR | 83 |
| Wang Feng et al., 2020 (2)-DTA/China | /Yes | National Health Commission of China/Yes | January 2020 and March 2020/28 days (median). | 108 | > 2.63 μg/mL | Mortality | ROC | DTA | 84 |
| Wang Kun et al., 2020/China | Cohort (consecutive)/Yes | National Health Commission of China (Version 5)/Yes | Jan 7 to Feb 11, 2020 | 296 | > 0.5 μg/mL? | Mortality | ULR, MLR | UNI and MULTI-OR for mortality. | 85 |
| Wang Miao et al., 2020/China | Retrospective/? | COVID-19 Diagnosis and Treatment Program 7th Edition (China)/ pharyngeal swab | February 10 to March 30, 2020 | 77 | Dynamic variation of D-D in the course of disease | Severity (Severe and Critical) | ? | UNI-OR for severity | 86 |
| Wang Tao et al., 2020/China | Retrospective-Nationwide multi-center/Yes | National Health Commission of China (Version 7)/? | By January 31, 2020 | 107 | > 1.5 mg/L | Severity (IMV requirement) | Events on either side of cut-off, MLR | UNI and MULTI-OR for severity | 87 |
| Wang Yafei et al., 2020/China | Retrospective/Yes | WHO interim guidance/Yes | January 1 to February 10, 2020 | 110 | Dimer/SD (3.25 μg/mL) | Severity (Severe vs. Non-severe) | binomial logistic  regression analysis | Independent-OR for severity | 88 |
| Watanabe Mikiko et al., 2020/Italy | Retrospective single-center (consecutive)/Yes | / naso- and oro-pharyngeal swabs | March 2020 | 150 | Based on dichotomic values on either side of the median. | Severity (admission to ICU, which includes requirement for intubation) | ULR, MLR | UNI-OR for severity | 89 |
| Wendel Garcia PD et al., 2020/Europe | Prospective observational cohort study (Europe-Multi-country)/Exempted | WHO/? | As  of April 22, 2020 (30 Days) | 639 | Log-D-D for Univariate and Multivariate Cox-PH models and ≥ 1560 µg/mL for K-M analysis | Mortality | Univariate & multivariable Cox proportional-hazard regression models Kaplan-Meier survival analysis. | UNI and MULTI-HR for mortality. | 90 |
| Xia Peng et al., 2020/China | Retrospective single-centre/Yes | Chinese National Health Commission (Version 5) | February 5 to March 20, 2020 | 81 | > 5 mg/ml FEU | Mortality | Cox proportional  hazards models | UNI-OR, UNI and MULTI-HR for Mortality | 91 |
| Xie Jianfeng et al., 2020/China | Retrospective Multi-centre/Yes | WHO, Diagnosis and management protocol of COVID-19 in China/Yes | January 1 to February 29, 2020/28 Days | 733 | High D-D level | Mortality | Multivariate  Cox proportional-hazards regression model |  | 92 |
| Xiong Bei et al., 2020/China | Retrospective/Yes | National  Health Commission of the People’s Republic of China (Version 6)/ Throat swab | As of March 21th, 2020 | 57 | > 500 ng/mL | Severity (Ordinary-svere-critical) & Mortality | Events on either side of the cut-off | UNI-OR for severity & Mortality | 93 |
| Yang Ai-Ping et al., 2020/China | Retrospective/? | WHO interim guidance/Yes | ? | 93 | 2.1 | Severity (Non-severe vs. severe) | ULR, ROC | UNI-OR for severity,  AUC | 94 |
| Yang Qing et al., 2020/China | Retrospective single-center/Yes | WHO interim guidance/Yes | January 1 to February  29, 2020. | 226 | ≥0.83 µg/mL | Mortality | Cox proportional hazard & Kaplan-Meier Analysis | MULTI-HR for Mortality | 95 |
| Yao Qingchun et al., 2020/China | Retrospective/Yes | WHO interim guidance/ Throat-swab or sputum | January 30, 2020 to February 11, 2020/As of March 3 (Median 19 Days). | 96 | > 1 µg/mL | Severity (Non-severe vs. severe), Mortality | Events on either side of cut-off, and Multivariate analysis | CALC-OR for severity and Mortality. MULTI-OR for D-D not reported. | 96 |
| Yao Yumeng et al., 2020/China | Retrospective (consecutive)/Yes | National health commission of China (Version 6)/Yes | January 28 to March 08, 2020. | 248 | > 2 mg/L | Severity and Mortality | Events on either side of cut-off. ULR, MLR, ROC | CALC-OR for severity. UNI and MULTI-OR for Mortality. DTA | 97 |
| Yu Caizheng et al., 2020/China | Retrospective/Yes | National commission of China (Version 5-6)/Throat Swab | January 14, 2020 and February 28, 2020 | 1484 | ≥ 0.5 mg/L | Severity (Severe vs. Non-severe) | Events on either side of cut-off, MLR | CALC-OR and MULTI-OR for Severity. | 98 |
| Yu Hai-Han et al., 2020/China | Retrospective/Yes | WHO/Yes | January 27 to March 5, 2020 | 1561 | > 0.5 μg/mL | Severity (Mild vs. severe) | MLR | MULTI-OR for severity | 99 |
| Zeng Da-Xiong et al., 2020/China | Retrospective single-center/Yes | WHO interim guidance/ pharyngeal or throat swab | February 9 to  March 9, 2020/28 Days | 274 | 0.68 µg/L (Median) | Mortality | MLR & Kaplan-Meier Analysis | MULTI-OR for mortality. | 100 |
| Zeng Zihang et al., 2020/China | Retrospective Multi-center/Yes | WHO interim guidance/ | January 22 to March 14, 2020/30 Days, until March 29, 2020. | 461 | Above reference value (Median 0.31 µg/mL). | Severity (ICU vs. Non-ICU) | Bivariate Cox proportional hazards regression model | Bivariate HR for severity | 101 |
| Zhan Ting et al., 2020/China | Retrospective/Yes | Chinese  National Health Committee (version 5-7)/ nasal or pharyngeal  swab | January 12 to March 8, 2020 | 405 | Increased D-D | Severity (Severe vs. Non-severe) | Increased D-D reported in both groups | UNI-OR for severity | 102 |
| Zhang Gemin et al., 2020/China | Retrospective/Yes | National health commission of China (Version 5)/ nasal and pharyngeal swab | January 16 to February 25, 2020/until  March 2, 2020. | 95 | > 1 mg/L | Severity, CEP (ICU care, MV, Death), Mortality | Events on either side of cut-off | CALC-OR for severity, CEP and Mortality | 103 |
| Zhang Jin-jin et al., 2020/China | Retrospective/Yes | National health committee of China (Version 3-5)/ pharyngeal  swab | January  16 to February 3, 2020 | 81 | > 0.243 µg/mL | Severity (Severe vs. Non-severe) | Events on either side of cut-off | CALC-OR for severity | 104 |
| Zhang Jin-Jin et al (2)., 2020 | Retrospective/Yes | National health committee of China (Version 3-5)/ pharyngeal  swab | as of March 28th, 2020 | 231 | > 0.5 µg/mL | Severity (Non-severe vs. severe alive), Mortality (Non-survivors vs. survivors) | Events on either side of cut-off, ULR, MLR | CALC-OR for severity and mortality. Age-adjusted OR for Mortality. | 105 |
| Zhang Jinping et al., 2020/China | Retrospective/? | WHO interim guidance/Yes | January 16, 2020 and February 20, 2020 | 19 | Increase in D-D (Median: 0.91 mg/L FEU). | Mortality | Events on either side of cut-off | CALC-OR | 106 |
| Zhang Litao et al., 2020/China | Retrospective/Yes | WHO interim guidance/Yes | January 12, 2020 to March 15, 2020/29 Days (Median) | 343 | ≥ 2 µg/mL FEU | Mortality | Events on either side of cut-off. Cox-proportional hazard models & Kaplan-Meier Analysis, ROC | CALC-OR and HR for Mortality, DTA | 107 |
| Zhang Shuxiang et al., 2020/China  CHINSESE | Retrospective single-center/ | New Coronavirus Infected Pneumonia Diagnosis and  Treatment Program (Trial Version 5)/ nasopharyngeal swabs | January 22 to February 4, 2020 | 34 | < 1 µg/L | Severity (Severe vs. non-severe) | Events on either side of cut-off. | CALC-OR for Severity | 108 |
| Zhao Jing et al., 2020/China | Retrospective/Yes | WHO interim guidance, Chinese National Health Commission (Version 5)/Yes | January 27 and  February 27, 2020/until April 1, 2020 | 29 | > 0.5 µg/L | Severity (Severe vs. non-severe) | Events on either side of cut-off. | CALC-OR for Severity | 109 |
| Zhong Zhe-Feng et al., 2020/China | Prospective/Yes | National Health  Commission of China (Version 7)/ pharyngeal swab | January 16 to March 2, 2020 | 43 | > 0.5 mg/L | Severity (Severe vs. non-severe) | Events on either side of cut-off. | CALC-OR for Severity | 110 |
| Zhou Fei et al., 2020/China | Retrospective multi-center/Yes | WHO interim guidance, Chinese management guideline for COVID-19  (version 6.0)/Yes | Dec 29, 2019 and Jan 31, 2020 | 127 | 0.5-≤ 1 µg/mL &  > 1 µg/mL | Mortality | ULR, MLR | UNI and MULTI-OR reported for Mortality. | 6 |
| Zou Ying et al., 2020/China | Retrospective/? | Diagnosis and Treatment Protocols for Novel Coronavirus Pneumonia (Version 7)/ | January 20 and February 24, 2020 | 303 | > 0.5 µg/mL | Severity (Severe vs. Non-severe) | Events on either side of cut-off. | CALC-OR for Severity | 111 |

ARDS=acute respiratory distress syndrome. ULR=univariate logistic regression. MLR=multivariate logistic regression. ROC=receiver operating curve, DTA=diagnostic test accuracy, OR=odds ratio. HR=hazards ratio.

***Appendix Table 2:*** The Studies excluded in Meta-analysis

| **Study/Country** | **Study type/**  **IEC** | **COVID-19 criteria/**  **RT-PCR** | **Duration/Follow-up** | **Total-(n) with D-dimer** | **Cut-off** | **Outcome** | **Reason for exclusion in Meta-analysis** | **Reference** |
| --- | --- | --- | --- | --- | --- | --- | --- | --- |
| Chen Qingxing et al., 2020/China | Retrospective/Yes | China's National  Health Commission (version 6)/ | Jan-Feb 2020 | 54 | NA | Severity (Severe vs. Critical) | No proper data suitable for meta-analysis | 112 |
| Francone Marco et al., 2020/Italy | Retrospective single-center/Yes | WHO, Chinese CDC/ nasopharyngeal swabs | March 6 to March 22, 2020/14.2 Days (range 1-24 Days) | 130 | > 500 ng/mL | Mortality | No proper data suitable for meta-analysis | 113 |
| Gavin Warren et al., 2020/USA | Retrospective single-center/reviewed-exempted | Confirmed COVID-19 cases/Yes | March 1-March 31, 2020/Data collection until June5, 2020. |  | > 575.5 ng/mL | Severity (MV) and Mortality | No proper confidence interval data suitable for meta-analysis | 114 |
| Guo Ting et al., 2020/China | Retrospective multi-center/? | WHO interim guidance, National Health  Commission of China (Version 7)/ | January 21 to February 19, 2020/ followed  up to March 11, 2020. | 105 | > 1 mg/L | Groups were compared based on age, not on severity or survival basis. | No proper data suitable for meta-analysis. Groups were compared based on age, not on severity or survival basis. | 115 |
| Huang Dong et al., 2020/China | Retrospective multi-center/Yes | Chinese Guidance for COVID-19 (7th edition)/Yes | January 21 to February 7,2020 |  | ≥ 0.5 mg/L | Case-control, study comparing suspected and vs. Confirmed cases | Case-control, study comparing suspected and vs. Confirmed cases | 116 |
| Li Xun et al., 2020/China | Retrospective single-center/Yes |  | Jan 14 to Feb 13, 2020 | 12 |  | Only dead patient group | No proper data suitable for meta-analysis. Only dead patient group. | 117 |
| Ma Simin et al., 2020/China | Retrospective?/Yes | WHO, National Health Commission of China (Version 5)/ nasal and pharyngeal swab | January 28, 2020 to February 29, 2020 | 93 | > 5 μg/mL | Mortality | No proper data suitable for meta-analysis Flu vs. Non-Flu design. | 118 |
| Martín-Rojas RM et al., 2020/Spain | Retrospective/Yes | Confirmed-COVID-19/ nasopharyngeal swab | April 3 and May 3, 2020 | 206 | > 250 ng/mL | Poor prognosis (ICU vs Non-ICU, Non-survivors vs. Survivors-Mortality) | No suitable data related to HR and CI for meta-analysis. | 119 |
| Wu Yiqun et al., 2020/China | Retrospective single-center/Yes | National health commission of China/ pharyngeal swab | February 10, 2020  and March 30, 2020 |  | ≥ 0.5 µg/mL | long-term hospitalization | No suitable data for meta-analysis. | 120 |
| Ye Wenjing et al., 2020/China | Retrospective single-center/exempted | National commission of China/Yes | January 1 and March 16, 2020 |  | Initial (> 0.73 mg/L), Peak (> 3.78 mg/L) | Mortality | No suitable data related to HR and CI for meta-analysis. | 121 |
| Zhang Bicheng et al., 2020/China | Retrospective/Yes | / nasal and pharyngeal swab | January 11, 2020 to February 10,  2020 | 68 | > 0.55 mg/L | Mortality | No suitable data for meta-analysis. | 122 |
| Zhou Yulong et al., 2020/China | Retrospective/Yes | National Health Commission of China (Version 5)/ | January 28 and February 6, 2020 |  | NA | Severity (Disease aggravation after admission) | No suitable data related to OR and CI for meta-analysis. | 123 |
| Wu Chaomin et al., 2020/China  EXCLUDE-Improper Data | Retrospective cohort/Yes | WHO interim guidance/ throat swab | December 25, 2019 and January 26, 2020/Up to February 13,  2020. | 201 | Above reference | Severity (ARDS) and Mortality | No suitable data related to HR and CI for meta-analysis. | 124 |

ARDS=acute respiratory distress syndrome. ULR=univariate logistic regression. MLR=multivariate logistic regression. ROC=receiver operating curve, DTA=diagnostic test accuracy, OR=odds ratio. HR=hazards ratio.

***Appendix Table 3:*** Quality assessment using QUIPS tool

| **Study** | **A** | **B** | **C** | **D** | **E** | **F** | **Overall-Risk of Bias** | **Remarks** | **Reference** |
| --- | --- | --- | --- | --- | --- | --- | --- | --- | --- |
| Aloisio Elena et al., 2020 | LOW | LOW | LOW | LOW | MODERATE | MODERATE | MODERATE | MLR performed only on 72 patients & Adjusted-OR for DD has not been reported. | 8 |
| Aloisio Elena et al., 2020 (2) | MODERATE | LOW | LOW | LOW | NA | MODERATE | MODERATE | ROC | 9 |
| Al-Samkari Hanny et al., 2020 | LOW | LOW | LOW | LOW | LOW | LOW | LOW | Adjusted OR reported | 10 |
| Ayanian Shant et al., 2020-OR | LOW | LOW | MODERATE | LOW | MODERATE | LOW | LOW | D-D available in 248 cases and adjusted for sex and COMS | 11 |
| Bao Changqian et al., 2020 | LOW | LOW | MODERATE | LOW | HIGH | MODERATE | MODERATE | D-D method not given. Adjusted-OR not reported | 12 |
| Bi Xiaojie et al., 2020 | LOW | LOW | LOW | LOW | MODERATE | MODERATE | LOW | D-D method not given. Adjusted-OR not reported | 16 |
| Bossini Nicola et al., 2020 | MODERATE | LOW | MODERATE | LOW | MODERATE | MODERATE | MODERATE | D-D method not given. Adjusted-OR not reported. | 17 |
| Cariou Bertrand et al., 2020 | LOW | LOW | MODERATE | LOW | MODERATE | LOW | LOW | D-D data available in 397 cases and Adjusted-OR not reported | 18 |
| Cecconi Maurizio et al., 2020 | LOW | LOW | LOW | LOW | MODERATE | MODERATE | LOW | D-D method not given. Adjusted-HR not reported | 19 |
| Cen Yuan et al., 2020-HR | LOW | LOW | LOW | LOW | LOW | LOW | LOW | MULTI-HR reported. | 20 |
| Chen Lei et al., 2020 | LOW | LOW | MODERATE | LOW | LOW | LOW | LOW | D-D in 1602 cases.  MULTI-HR reported. | 21 |
| Chen Liu Ying et al., 2020 | LOW | LOW | LOW | LOW | LOW | LOW | LOW | MULTI-HR reported. | 22 |
| Chen Qingxing et al., 2020 | MODERATE | NR | MODERATE | MODERATE | LOW | HIGH | HIGH | No proper data suitable for meta-analysis. | 112 |
| Chen Ruchong et al., 2020 | LOW | NR | LOW | LOW | MODERATE | MODERATE | MODERATE | No-HR values reported for D-D | 23 |
| Chen Tao et al., 2020 | LOW | LOW | MODERATE | LOW | MODERATE | LOW | MODERATE | D-D in 247 cases. | 24 |
| Cheng Anying et al., 2020 | LOW | LOW | LOW | LOW | LOW | LOW | LOW |  | 25 |
| Chilimuri Sridhar et al., 2020 | LOW | MODERATE | LOW | LOW | LOW | LOW | LOW |  | 26 |
| Cummings Matthew et al., 2020 | LOW | LOW | LOW | LOW | LOW | LOW | LOW |  | 28 |
| Di Micco et al., 2020 | HIGH | NR | MODERATE | MODERATE | HIGH | MODERATE | HIGH | Case-control study | 30 |
| Dong Yalan et al., 2020 | LOW | NR | LOW | LOW | LOW | LOW | LOW | Adjusted-OR reported | 31 |
| Duan Jun et al., 2020 | LOW | LOW | LOW | LOW | NA | LOW | LOW | DTA | 32 |
| Francone Marco et al., 2020 | LOW | NR | LOW | LOW | MODERATE | HIGH | HIGH | No proper data suitable for meta-analysis | 113 |
| Gao Yong et al., 2020 | MODERATE | NR | LOW | LOW | MODERATE | MODERATE | HIGH | Independent-OR, DTA | 34 |
| Gavin Warren et al., 2020 | LOW | LOW | LOW | LOW | HIGH | MODERATE | HIGH | No proper OR and confidence interval data suitable for meta-analysis | 114 |
| Ghaffari Rahbar M et al., 2020 | HIGH | NR | LOW | LOW | HIGH | MODERATE | HIGH | Less sample size of 19 kidney transplant cases with COVID-19.  Adjusted-OR not reported. | 35 |
| Guan Wei-jie et al., 2020 | LOW | LOW | LOW | LOW | MODERATE | LOW | LOW | D-D values in 560of 1099 cases. No adjusted-OR reported. | 4 |
| Guo Ting et al., 2020 | LOW | NR | HIGH | HIGH | HIGH | HIGH | HIGH | No proper data suitable for meta-analysis. Groups were compared based on age. | 115 |
| Harmouch Farah et al., 2020 | MODERATE | NR | MODERATE | LOW | LOW | LOW | MODERATE | No-PCR Information. D-D values in 420 cases. | 38 |
| Huang Dong et al., 2020 | LOW | NR | MODERATE | LOW | LOW | LOW | MODERATE | No suitable data. Case-control, study comparing suspected and vs. Confirmed cases | 116 |
| Huang Hong et al., 2020 | LOW | NR | HIGH | HIGH | HIGH | HIGH | HIGH | D-D values in 146 cases. COVID-19 with and without ILD. Adjusted-OR not reported. | 39 |
| Li Chenghong et al., 2020 | LOW | NR | LOW | LOW | MODERATE | MODERATE | MODERATE | Initial D-D values in 749 cases and Day-3 levels in 598 cases. MULTI-OR not reported. | 7 |
| Li Kaiyan et al., 2020 | LOW | NR | LOW | LOW | MODERATE | MODERATE | MODERATE | MULTI-OR not reported. | 42 |
| Li Qiubai et al., 2020 | LOW | LOW | LOW | LOW | LOW | LOW | LOW | Baseline D-D values in 1239 and Max D-D in 1262 cases.  MULTI-OR reported. | 43 |
| Li Tao et al., 2020 | LOW | LOW | MODERATE | LOW | LOW | LOW | MODERATE | MULTI-OR reported. | 44 |
| Li Xun et al., 2020 | HIGH | NA | HIGH | HIGH | MODERATE | MODERATE | HIGH | No proper data suitable for meta-analysis. Only dead patient group. | 117 |
| Li Yong et al., 2020 | LOW | LOW | LOW | LOW | LOW | LOW | LOW | Initial and dynamic D-D prognostic UNI and MULTI-OR reported. | 45 |
| Li Yumin et al., 2020 | LOW | LOW | LOW | LOW | LOW | LOW | LOW | UNI and MULTI-OR reported | 46 |
| Liao Danying et al., 2020 | LOW | LOW | LOW | LOW | LOW | LOW | LOW | D-D values missing in 47. MULTI-OR reported | 5 |
| Liu Fengjun et al., 2020 | LOW | LOW | LOW | LOW | LOW | LOW | LOW | Initial and dynamic D-D and MULTI-HR with AUC reported. | 47 |
| Liu Jiao et al., 2020 | LOW | LOW | LOW | LOW | LOW | LOW | LOW | MULTI-OR has been reported | 48 |
| Liu Jingmei et al., 2020 | LOW | LOW | LOW | MODERATE | LOW | LOW | LOW | MULTI-OR reported for CEP | 49 |
| Liu Q et al., 2020 | LOW | LOW | LOW | LOW | LOW | LOW | LOW | MULTI-OR reported for Deaths. DTA | 50 |
| Liu Qin et al., 2020 | LOW | NR | MODERATE | LOW | LOW | LOW | LOW | MULTI-OR reported for CEP | 51 |
| Liu Sheng-Ping et al., 2020 | LOW | LOW | LOW | MODERATE | LOW | LOW | LOW | MULTI-OR reported for CEP | 52 |
| Liu Tao et al., 2020 | MODERATE | LOW | NR | LOW | MODERATE | LOW | MODERATE | MULTI-OR not reported | 53 |
| Liu Xiaoyan et al., 2020 | LOW | NR | LOW | LOW | MODERATE | MODERATE |  | MULTI-OR not reported | 54 |
| Long Hui et al., 2020 | LOW | LOW | LOW | LOW | HIGH | MODERATE | HIGH | No comorbidities and MULTI-OR reported | 55 |
| Ma Simin et al., 2020 | LOW | NR | HIGH | HIGH | MODERATE | MODERATE | HIGH | No proper data suitable for meta-analysis Flu vs. Non-Flu design.No MULTI-OR reported. | 118 |
| Maeda Tetsuro et al., 2020 | LOW | NR | MODERATE | LOW | LOW | MODERATE | MODERATE | MLR for D-D involved 149 cases.  MULTI-OR reported for IMV and Mortality. | 64 |
| Mikami Takahisa et al., 2020 | LOW | LOW | LOW | LOW | LOW | LOW | LOW | D-D levels in 2984 cases. UNI and MULTI-HR reported. | 65 |
| Moreno-Pérez Oscar et al., 2020 | MODERATE | NR | LOW | LOW | LOW | LOW | MODERATE | D-D levels in 63 cases for reported MULTI-OR. | 66 |
| Pan Feng et al., 2020 | MODERATE | NR | LOW | LOW | LOW | MODERATE | MODERATE | MULTI-OR not reported. | 67 |
| Paranjpe Ishan et al., 2020 | LOW | MODERATE | MODERATE | LOW | MODERATE | LOW | MODERATE | D-D in 399 cases. MULTI-OR not reported. | 68 |
| Petrilli Christopher et al., 2020 | LOW | LOW | MODERATE | LOW | LOW | LOW | LOW | D-D missing in 373 cases of of 2729. MULTI-OR for CEP and MULTI-HR for mortality were reported. | 69 |
| Qin Wei et al., 2020 | LOW | NR | LOW | LOW | LOW | LOW | LOW | Adjusted-Or for mortality reported. | 70 |
| Rocio Laguna-Goya et al., 2020 | LOW | LOW | LOW | LOW | MODERATE | LOW | LOW | Adjusted-OR not reported | 71 |
| Sciascia Savino et al., 2020 | LOW | NR | MODERATE | LOW | LOW | MODERATE | MODERATE | Adjusted HR-for mortality reported | 73 |
| Smadja David et al., 2020 | MODERATE | LOW | LOW | LOW | MODERATE | HIGH | HIGH | Events on either side of the cut-off. Adjusted-OR not reported | 76 |
| Somers EC et al., 2020 | LOW | NR | MODERATE | LOW | MODERATE | LOW | MODERATE | COVID-19 cases of TCZ and Non-TCZ groups. D-D in 129 of 158 cases. UNI and Bivariate OR and HR were reported for 28-Day CEP and Mortality. | 77 |
| Sun Ying et al., 2020 | MODERATE | NR | NR | LOW | LOW | LOW | MODERATE | MULTI-OR reported for severity. | 78 |
| Tang Ning et al., 2020 | LOW | LOW | LOW | LOW | LOW | LOW | LOW | MULTI-OR reported for Mortality | 79 |
| Tian Jianbo et al., 2020 | LOW | LOW | MODERATE | LOW | LOW | LOW | LOW | 232 COVID-19 patients with Cancer. D-D in 167 cases.  MULTI-OR for severity reported. | 81 |
| Volo T et al., 2020 | MODERATE | LOW | MODERATE | LOW | MODERATE | HIGH | HIGH | No MULTI-OR value has been reported. Only a p-value (0.028) is found. | 82 |
| Wang Feng et al., 2020 | LOW | NR | LOW | LOW | LOW | LOW | LOW | Events on either side of the cut-off. MULTI-OR not reported. | 83 |
| Wang Feng et al., 2020 (2) | LOW | LOW | LOW | LOW | NA | LOW | LOW | ROC | 84 |
| Wang Kun et al., 2020 | LOW | LOW | LOW | LOW | LOW | LOW | LOW | MULTI-OR for mortality reported. | 85 |
| Wang Tao et al., 2020 | LOW | NR | MODERATE | LOW | LOW | MODERATE | MODERATE | D-D in 107 cases. MULTI-OR reported | 87 |
| Watanabe Mikiko et al., 2020 | LOW | NR | LOW | MODERATE | LOW | MODERATE | MODERATE | MLR performed but no MULTI-OR reported. | 89 |
| Wu Chaomin et al., 2020 | LOW | LOW | MODERATE | LOW | MODERATE | MODERATE | MODERATE | D-D in 189 cases. No suitable data related to HR and CI for meta-analysis. | 124 |
| Wu Yiqun et al., 2020 | LOW | NR | HIGH | HIGH | MODERATE | MODERATE | HIGH | No suitable data for meta-analysis. | 120 |
| Yang Ai-Ping et al., 2020 | LOW | NR | LOW | MODERATE | HIGH | LOW | HIGH | Adjusted-OR not reported. | 94 |
| Yang Qing et al., 2020 | LOW | NR | LOW | LOW | LOW | LOW | LOW | MULTI-HR for mortality reported. | 95 |
| Yao Qingchun et al., 2020 | LOW | LOW | LOW | LOW | MODERATE | MODERATE | MODERATE | Events on either side of cut-off. MULTI-OR not reported. | 96 |
| Yao Yumeng et al., 2020 | LOW | LOW | LOW | LOW | LOW | LOW | LOW | MULTI-OR reported for mortality. | 97 |
| Ye Wenjing et al., 2020 | LOW | NR | LOW | LOW | LOW | HIGH | HIGH | MULTI-HR reported for Mortality (No proper and CI for HR) | 121 |
| Yu Caizheng et al., 2020 | LOW | LOW | MODERATE | LOW | LOW | LOW | LOW | D-D in 1484 cases. MULTI-OR reported. | 98 |
| Zeng Da-Xiong et al., 2020 | LOW | LOW | MODERATE | LOW | LOW | LOW | LOW | MULTI-OR for mortality reported | 100 |
| Zeng Zihang et al., 2020 | LOW | LOW | MODERATE | LOW | MODERATE | MODERATE | MODERATE | D-D in 451 cases. Bivariate-HR reported. MULTI-HR for severity not reported. | 101 |
| Zhang Bicheng et al., 2020 | LOW | NR | MODERATE | LOW | MODERATE | MODERATE | HIGH | No proper data | 122 |
| Zhang Gemin et al., 2020 | LOW | LOW | LOW | LOW | MODERATE | MODERATE | MODERATE | Events on either side of cut-off. MULTI-OR not reported. | 103 |
| Zhang Jin-jin et al., 2020 | LOW | NR | MODERATE | LOW | MODERATE | HIGH | HIGH | D-D in 81 cases. MULTI-OR not reported. | 104 |
| Zhang Jin-Jin et al (2)., 2020 | LOW | LOW | MODERATE | LOW | LOW | LOW | LOW | D-D in 231 cases. Events on either side of cut-off. MULTI-OR not reported. | 105 |
| Zhang Jinping et al., 2020 | MODERATE | LOW | HIGH | LOW | MODERATE | MODERATE | HIGH | Events on either side of cut-off. MULTI-OR not reported. | 106 |
| Zhang Litao et al., 2020 | LOW | LOW | LOW | LOW | LOW | LOW | LOW | MULTI-HR reported for Mortality. DTA | 107 |
| Zhang Shuxiang et al., 2020 | LOW | LOW | MODERATE | MODERATE | HIGH | MODERATE | HIGH | Events on either side of cut-off.MULTI-OR not reported. | 108 |
| Zhao Jing et al., 2020 | LOW | LOW | MODERATE | LOW | HIGH | MODERATE | HIGH | Events on either side of cut-off.MULTI-OR not reported. | 109 |
| Zhong Zhe-Feng et al., 2020 | LOW | NR | MODERATE | LOW | MODERATE | MODERATE | MODERATE | Events on either side of cut-off.MULTI-OR not reported. | 110 |
| Zhou Fei et al., 2020 | LOW | LOW | MODERATE | LOW | LOW | LOW | LOW | D-D reported in 172 cases. MULTI-OR reported for Mortality. | 6 |
| Zhou Yulong et al., 2020 | MODERATE | LOW | MODERATE | MODERATE | MODERATE | MODERATE | HIGH | Binary-OR for severity with no proper CI | 123 |
| Zou Ying et al., 2020 | LOW | LOW | LOW | LOW | MODERATE | LOW | LOW | MULTI-OR not reported. | 111 |
| Studies updated after August 5, 2020 | | | | | | | | | |
| Martín-Rojas RM et al., 2020 | LOW | NR | LOW | MODERATE | MODERATE | HIGH | HIGH | No proper HR and CI. | 119 |
| Baycan OF et al., 2020 | LOW | LOW | MODERATE | LOW | LOW | LOW | LOW | MULTI-OR reported for Mortality | 13 |
| Coppelli Alberto et al., 2020 | LOW | LOW | MODERATE | LOW | LOW | MODERATE | MODERATE | MULTI-HR not reorted. | 27 |
| Feng Xiaobo et al., 2020 | LOW | LOW | LOW | LOW | LOW | LOW | LOW | UNI & MULTI-HR for CEP | 33 |
| Gormez Selcuk et al., 2020 | LOW | LOW | MODERATE | LOW | LOW | LOW | LOW | MULTI-OR for CEP reported. D-D in 217 cases of 247. | 36 |
| Hanif Ahmad et al., 2020-OR | LOW | LOW | LOW | LOW | MODERATE | MODERATE | LOW | UNI-OR reported for severity and Mortality. Adjusted-OR not reported. | 37 |
| Li Chenze et al., 2020 | LOW | LOW | LOW | LOW | LOW | LOW | LOW | Initial D-D in 1970 cases, Dynamic D-D in 817 cases. UNI-OR for severity & Mortality. MULTI-OR for Mortality (Initial & dynamic/longitudinal). | 40 |
| Li Guozhen et al., 2020 | LOW | LOW | LOW | LOW | LOW | LOW | LOW | MULTI-OR reported for Mortality | 41 |
| Naymagon Leonard et al., 2020 | LOW | LOW | LOW | LOW | LOW | LOW | LOW | UNI & MULTI-HR for Intubation and Mortality. Multivariate analysis in 997 cases. | 29 |
| Sardu Celestino et al., 2020 | LOW | LOW | LOW | LOW | LOW | LOW | LOW | MULTI-HR for mortality reported. | 72 |
| Shang Yufeng et al., 2020 | LOW | LOW | LOW | LOW | LOW | MODERATE | LOW | UNI & MULTI-OR for Mortality. | 74 |
| Sisó-Almirall Antoni et al., 2020 | LOW | NR | MODERATE | LOW | LOW | LOW | MODERATE | D-D values in 165 of 322 cases. MULTI-OR for CEP reported. | 75 |
| Tao Zheying et al., 2020 | LOW | NR | MODERATE | LOW | LOW | LOW | MODERATE | 152 of 222 cases had D-D values. | 80 |
| Wang Miao et al., 2020 | LOW | NR | MODERATE | LOW | MODERATE | MODERATE | HIGH | Unadjusted-OR for relationship between D-D variations and severity reported. | 86 |
| Wendel Garcia PD et al., 2020 | LOW | LOW | MODERATE | LOW | LOW | LOW | LOW | UNI-HR based on D-D in 227 cases. | 90 |
| Xia Peng et al., 2020 | LOW | LOW | LOW | LOW | LOW | LOW | LOW | MULTI-HR reported for Mortality | 91 |
| Xie Jianfeng et al., 2020 | LOW | LOW | MODERATE | LOW | LOW | LOW | LOW | D-D values missing in 96 of 733 cases. MULTI-HR reported. | 92 |
| Xiong Bei et al., 2020 | LOW | LOW | MODERATE | LOW | MODERATE | MODERATE | MODERATE | Events on either side of the cut-off reported. UNI-OR for severity & Mortality. | 93 |
| Yu Hai-Han et al., 2020 | LOW | LOW | MODERATE | LOW | LOW | LOW | LOW | MULTI-OR for severity reported. | 99 |
| Zhan Ting et al., 2020 | LOW | NR | LOW | LOW | MODERATE | MODERATE | MODERATE | Increased D-D reported in both groups. MULTI-OR not reported for severity. | 102 |
| Berger JS et al., 2020 | LOW | LOW | LOW | LOW | LOW | LOW | LOW | MULTI-OR reported for CEP & Mortality | 14 |
| Bhadade Rakesh et al., 2020 | LOW | LOW | MODERATE | LOW | LOW | LOW | LOW | Independent-OR for Mortality reported. | 15 |
| Wang Yafei et al., 2020 | LOW | LOW | MODERATE | LOW | LOW | MODERATE | MODERATE | Adjusted-OR reported for Severity | 88 |

A=study participation, B=study attrition, C=prognostic factor measurement, D=outcome assessment, E=confounding, F=statistics

**Forest plots (Initial-D-dimer)**

***Appendix Figure 1:*** Pooled unadjusted-OR for disease severity in patients with COVID-19


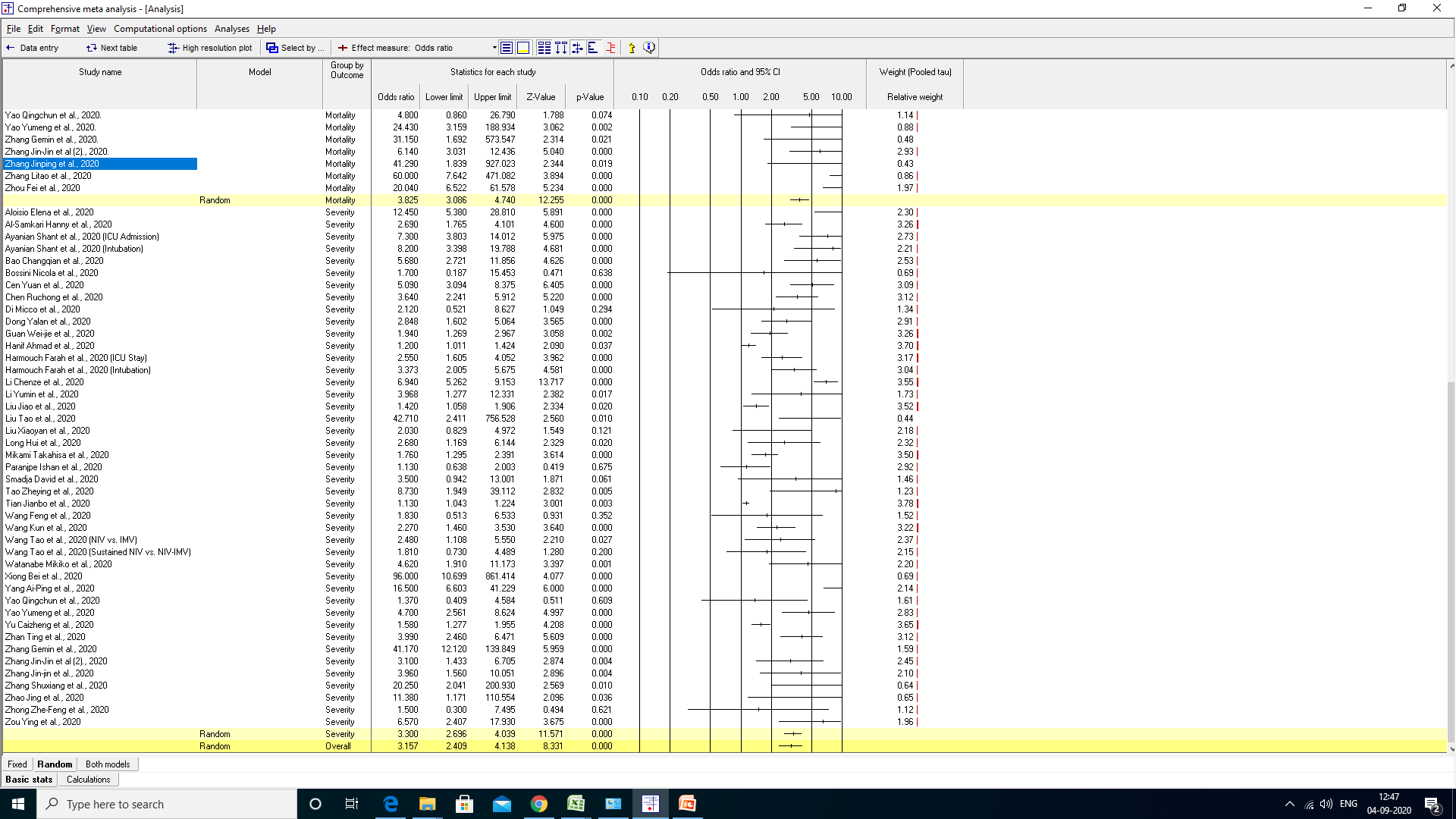


***Appendix Figure 2:*** Pooled unadjusted-OR for mortality outcomes in patients with COVID-19

**
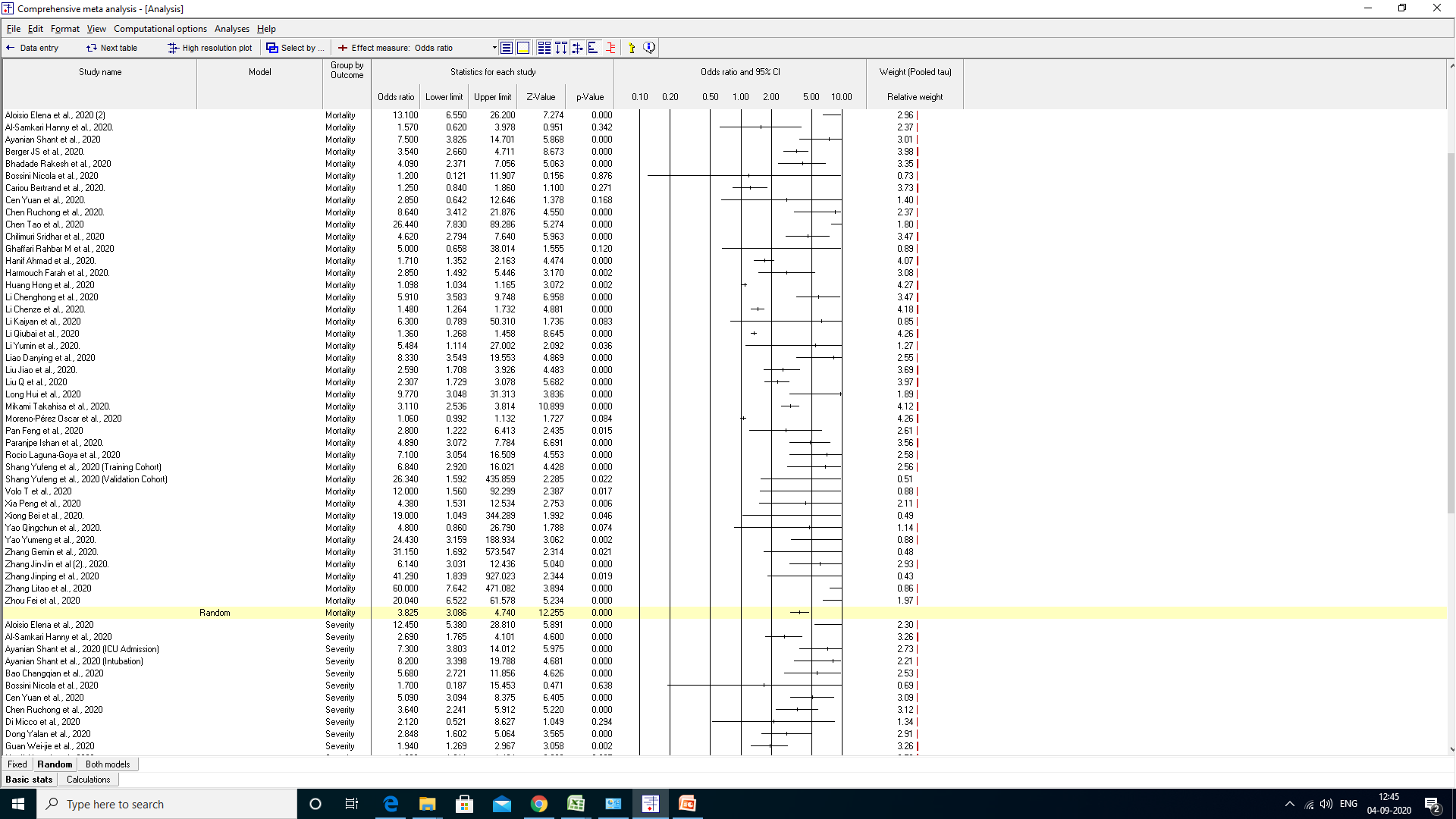
**

***Appendix Figure 3:*** Pooled unadjusted-OR for composite outcomes in patients with COVID-19

**
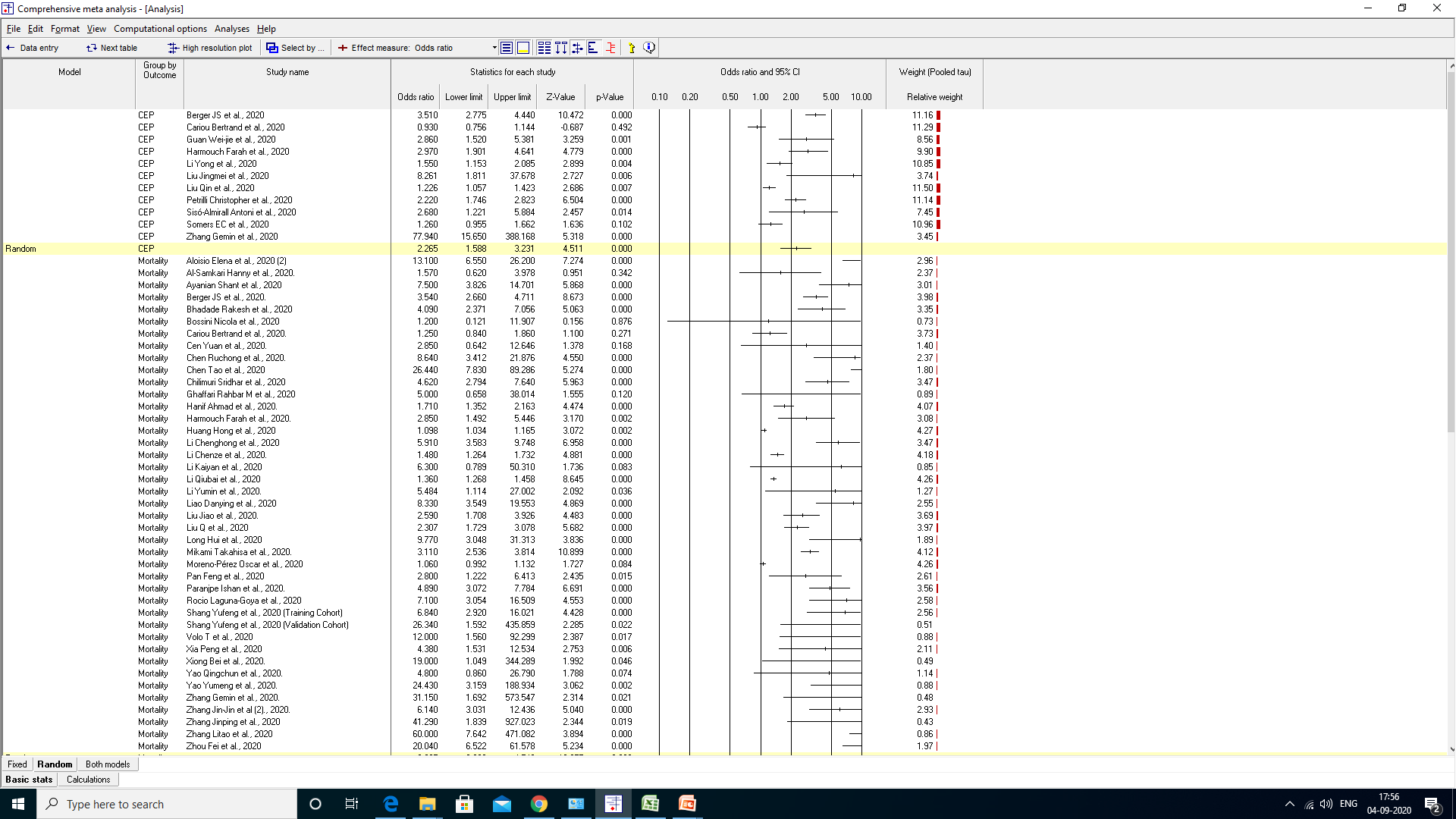
**

***Appendix Figure 4:*** Pooled adjusted-OR for disease progression (overall and outcome-wise) in patients with COVID-19


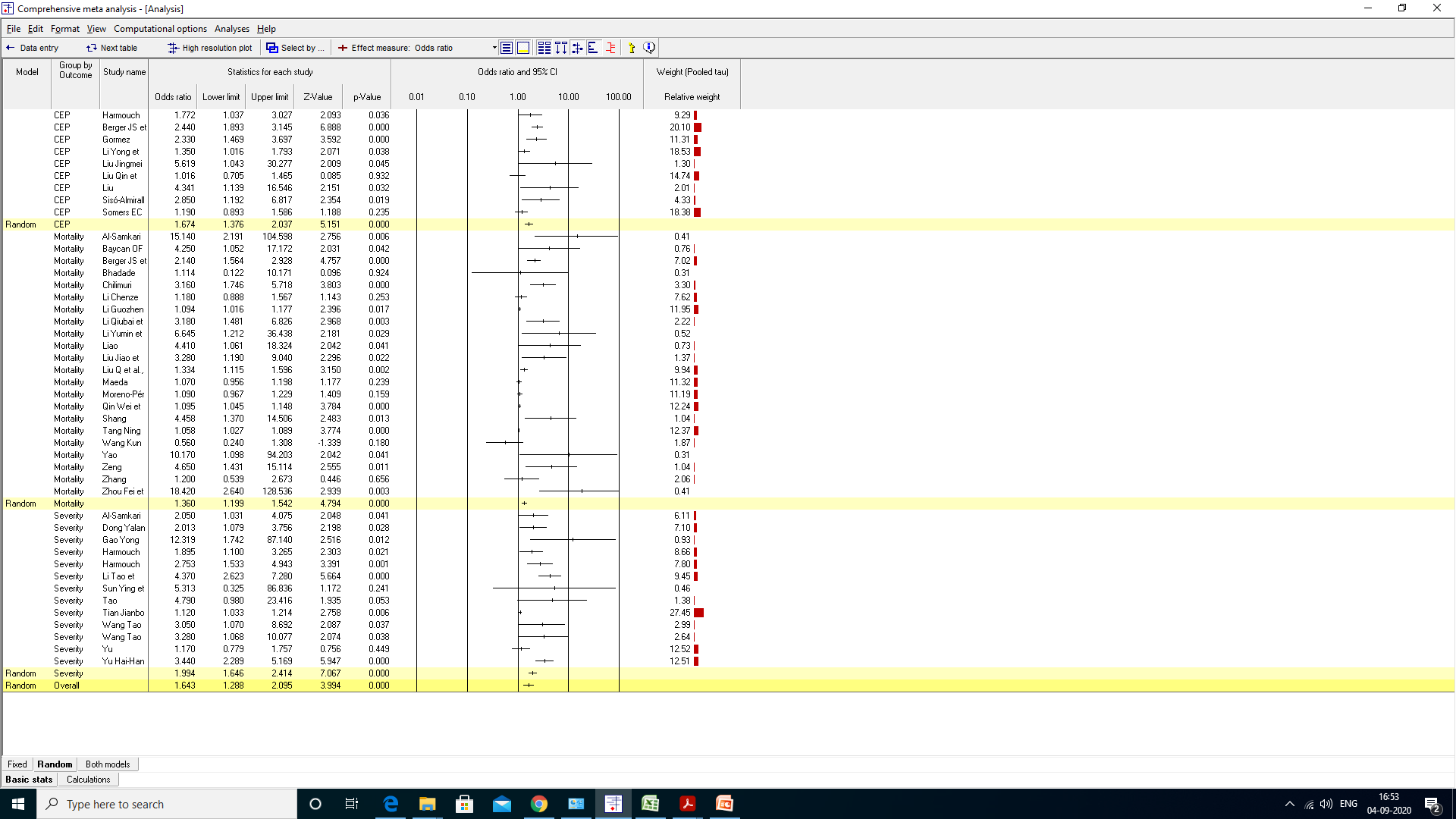


**Forest plots (Dynamic-D-dimer)**

***Appendix Figure 5:*** Pooled unadjusted-OR for disease progression (overall and outcome-wise for dynamic D-dimer) in patients with COVID-19

**
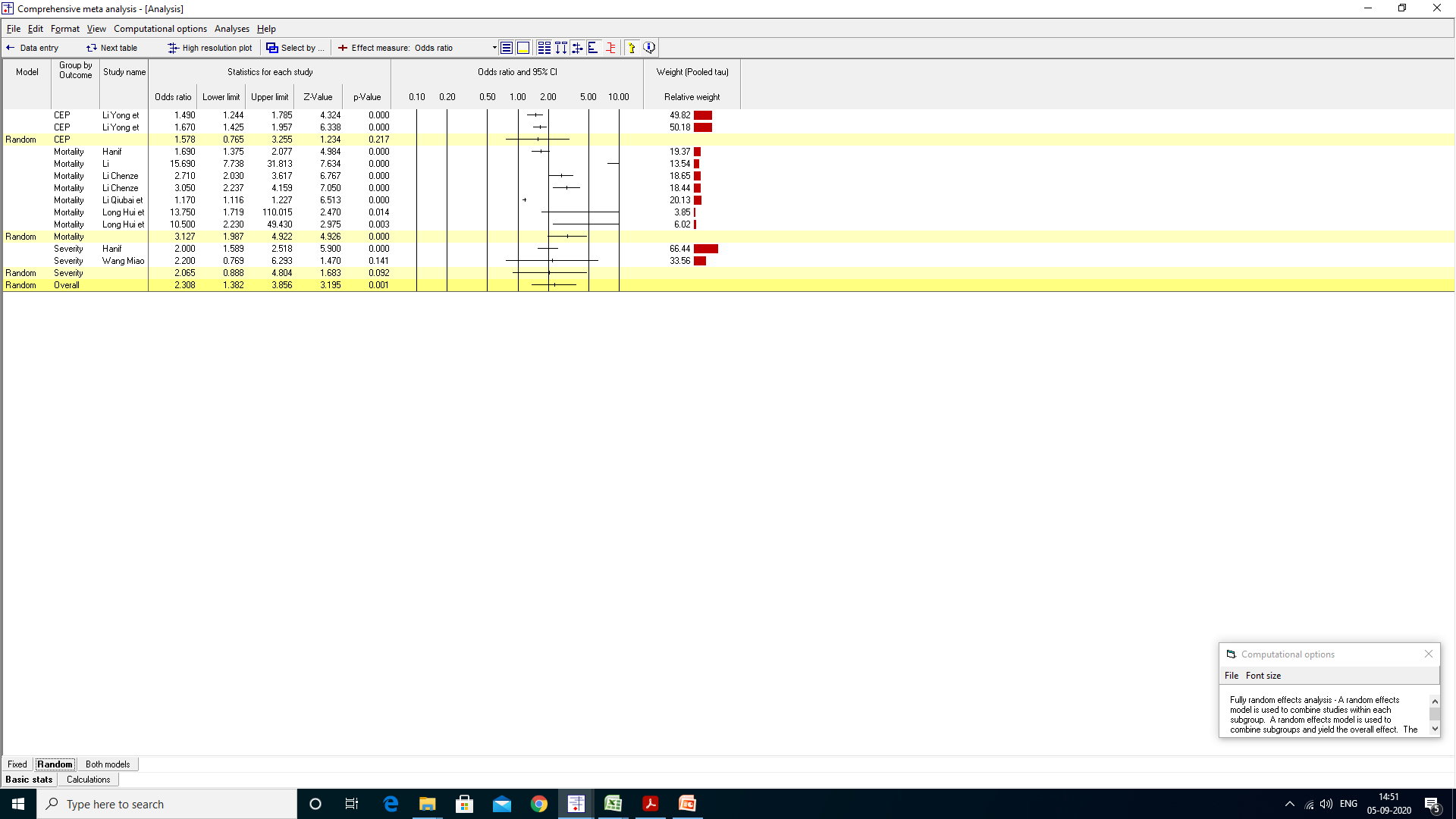
**

***Appendix Figure 6:*** Pooled adjusted-OR for disease progression (overall and outcome-wise for dynamic D-dimer) in patients with COVID-19


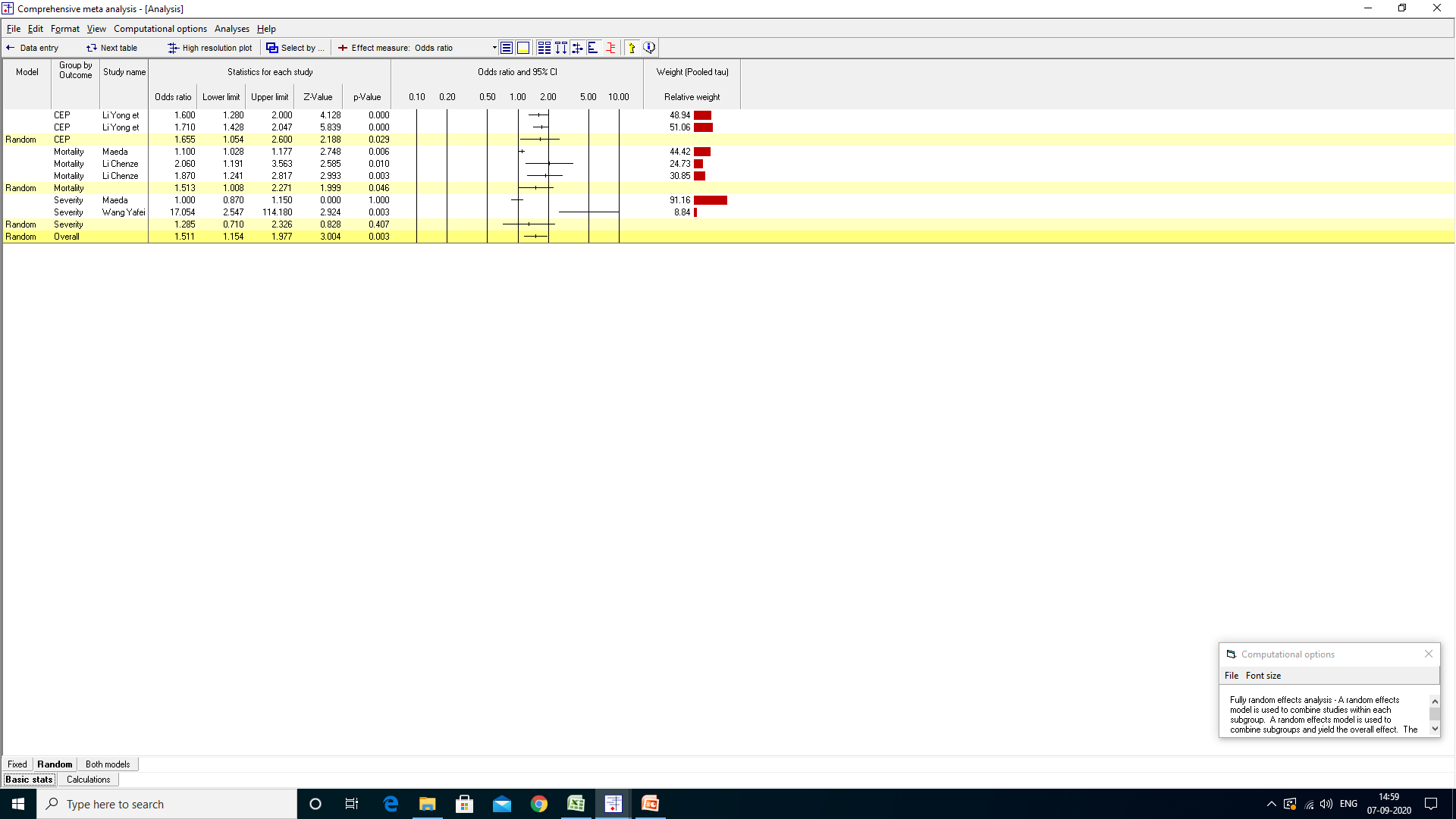


**Forest plots (Initial-D-dimer), Time-to-event Hazards Ratios**

***Appendix Figure 7:*** Pooled unadjusted-HR for disease progression (overall and outcome-wise for initial D-dimer) in patients with COVID-19

**
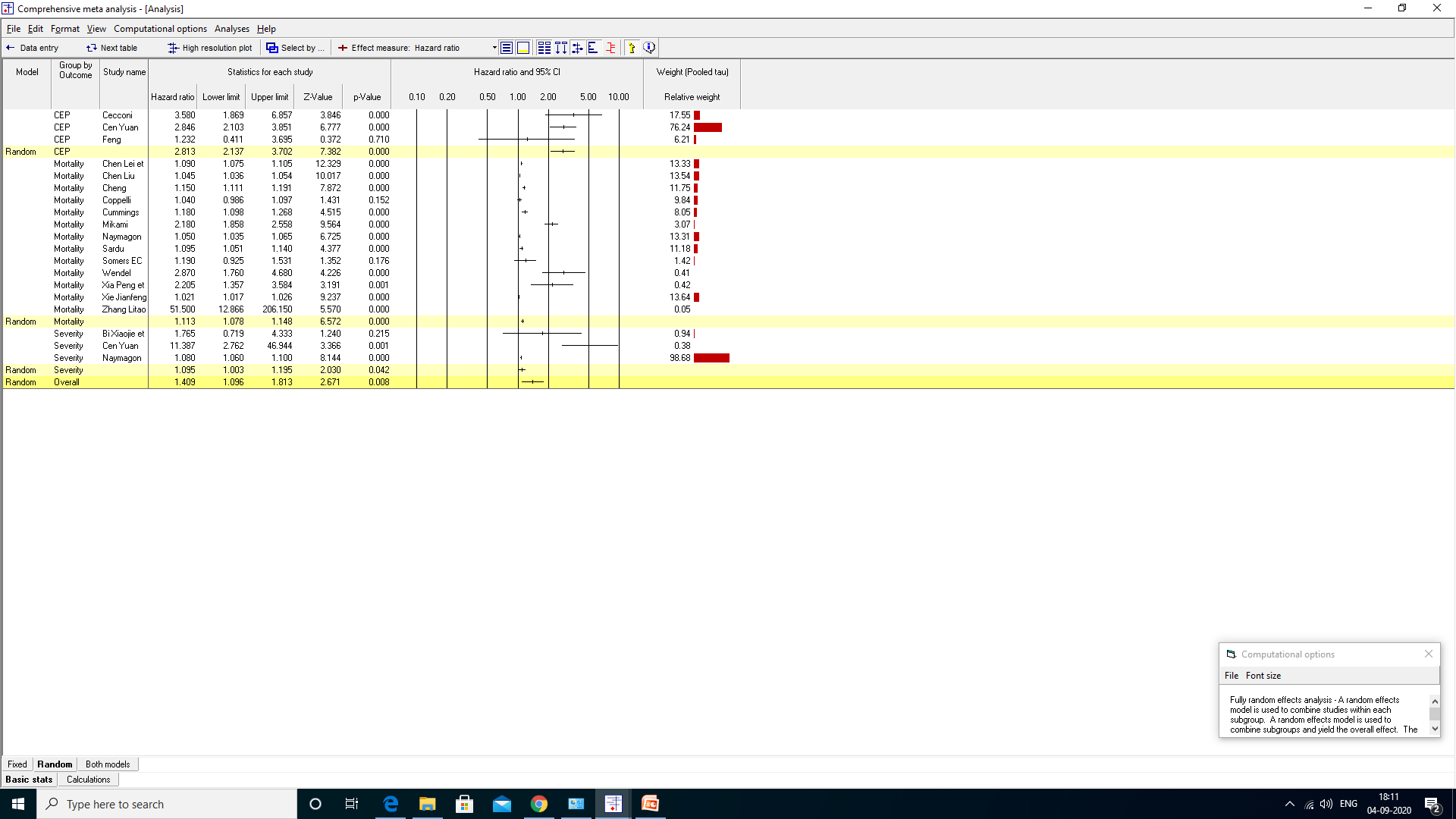
**

***Appendix Figure 8:*** Pooled adjusted-HR for disease progression (overall and outcome-wise for initial D-dimer) in patients with COVID-19


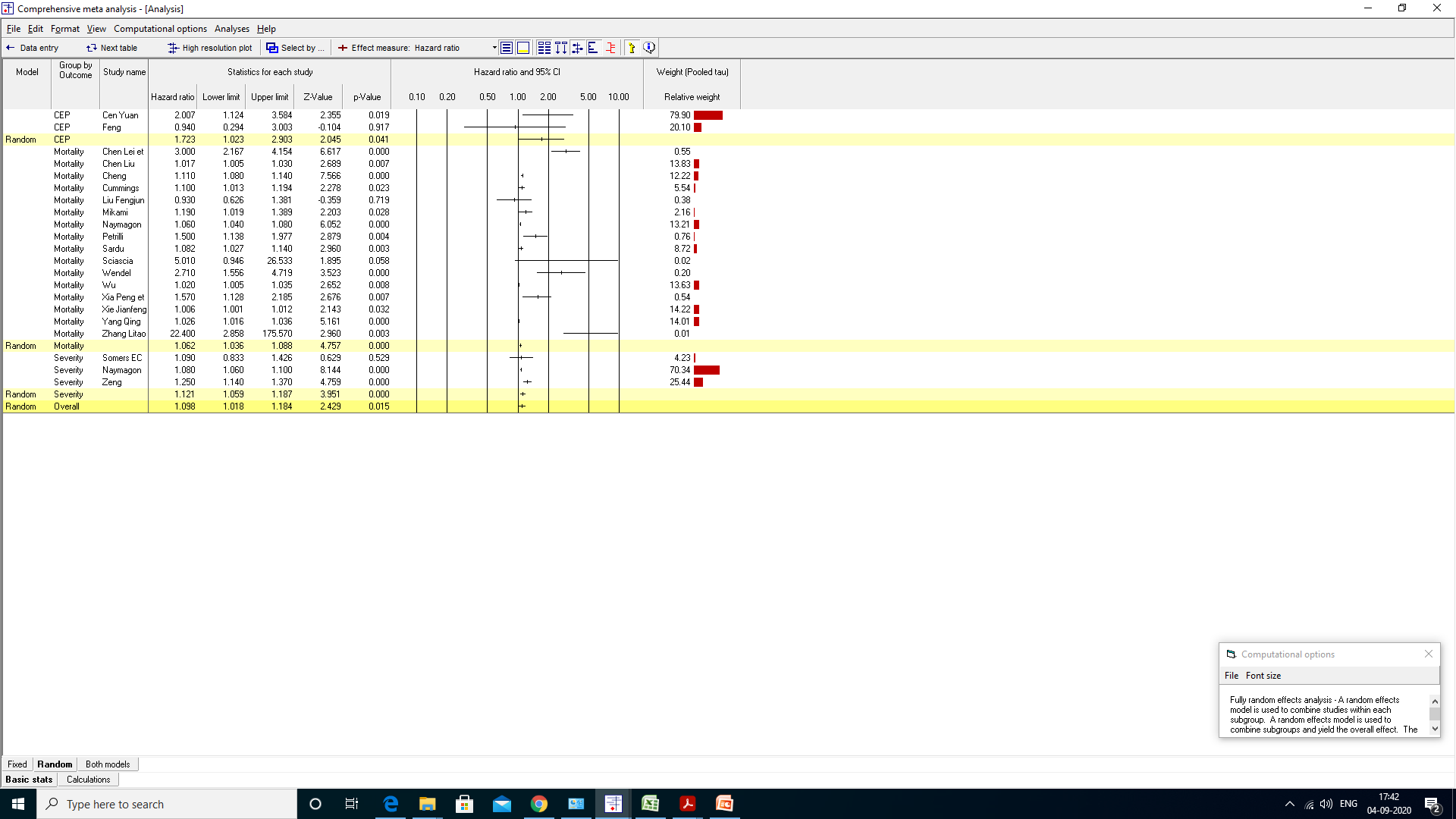


**Forest plots (Initial-D-dimer), Country-wide Sub-groups**

***Appendix Figure 9:*** Subgroup analysis based on ‘Country’ for the association of initial D-dimer with disease progression in COVID-19. Unadjusted-OR (A). Adjusted-OR (B)


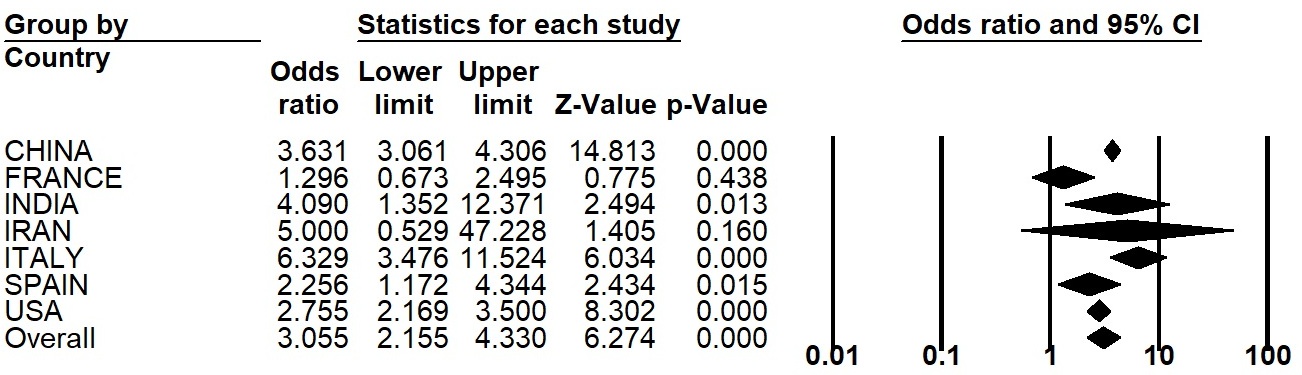


**B**


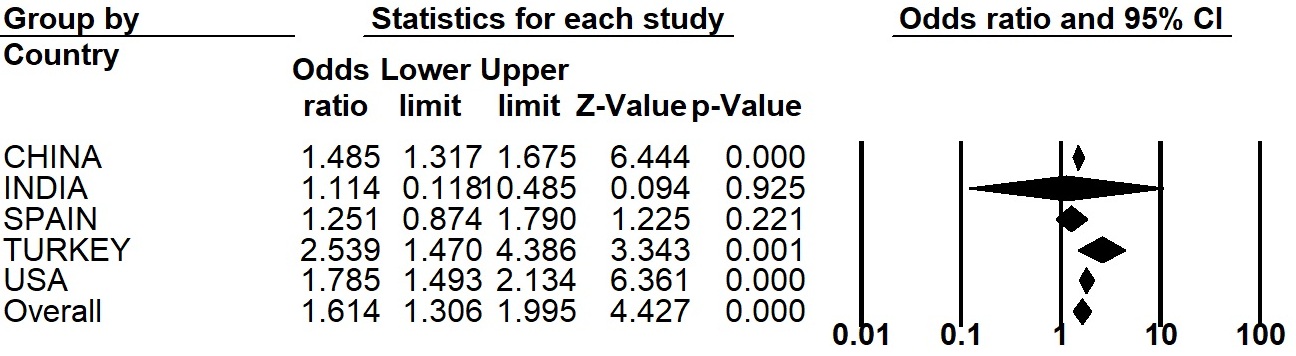


**Forest plots (Initial-D-dimer), Time-to-event Hazards Ratios, Country-wide Sub-groups**

***Appendix Figure 10:*** Subgroup analysis based on ‘Country’ for the association of initial D-dimer with disease progression in COVID-19. Unadjusted-HR (A). Adjusted-HR (B)

**
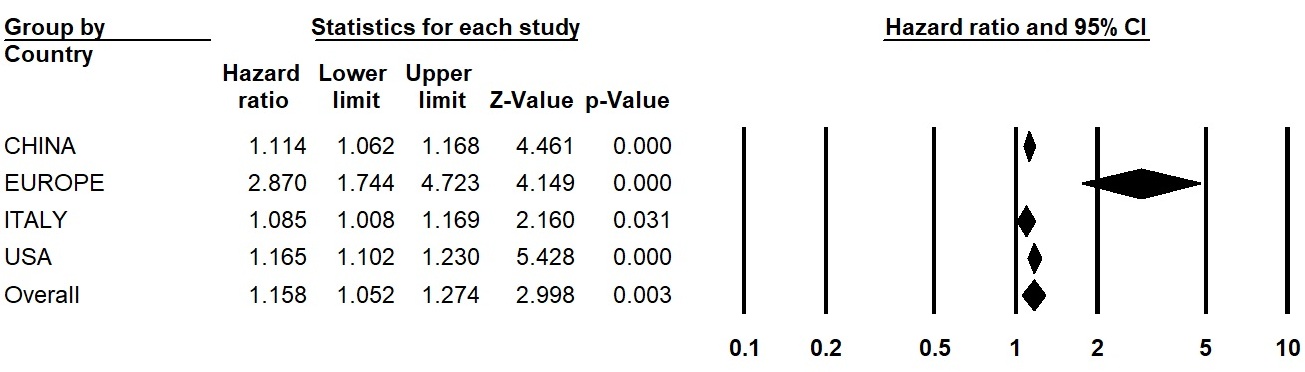
**

**B**

**
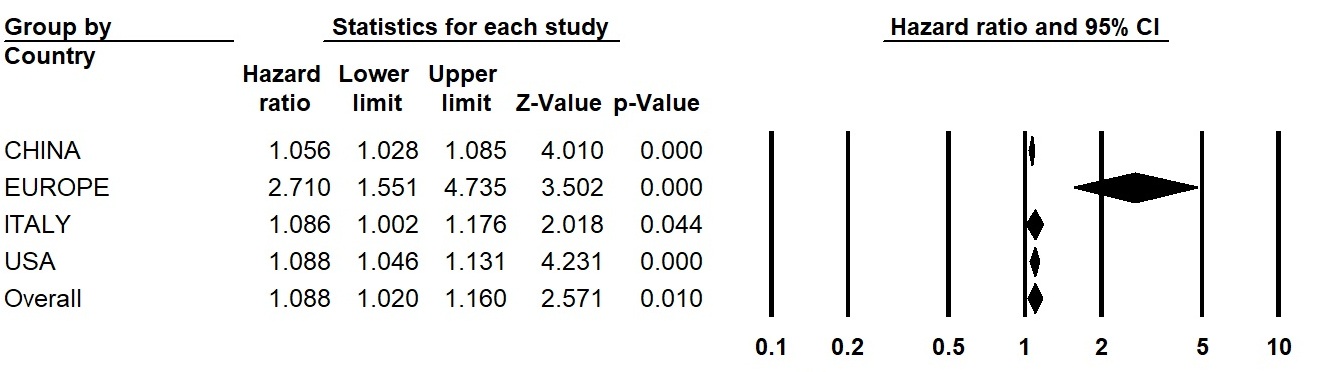
**
